# Development and validation of an interpretable machine-learning model for enteral nutrition-associated diarrhea in critically ill patients with ischemic stroke: a retrospective cohort study

**DOI:** 10.3389/fnut.2026.1890164

**Published:** 2026-07-14

**Authors:** Yang He, Dan Jin, Ming Liu, Jinglan Liu

**Affiliations:** 1 Department of Critical Care Medicine, The First College of Clinical Medical Science, Yichang Central People's Hospital, China Three Gorges University, Yichang, Hubei, China; 2 Yichang Central People's Hospital, Yichang, Hubei, China

**Keywords:** enteral nutrition-associated diarrhea, intensive care unit, ischemic stroke, machine learning, nursing risk prediction, random forest, SHAP

## Abstract

**Background:**

Enteral nutrition-associated diarrhea (ENAD) is a frequent gastrointestinal complication during enteral nutrition (EN) in critically ill patients. In ICU patients with ischemic stroke, neurological impairment, dysphagia, reduced consciousness, immobility, infection, and intensive care interventions may further impair gastrointestinal tolerance; however, disease-specific prediction tools are scarce.

**Objective:**

To develop and internally validate an interpretable machine-learning model for individualized ENAD prediction in ICU patients with ischemic stroke receiving EN.

**Methods:**

This single-center retrospective cohort study included adult ICU patients with ischemic stroke who received EN at Yichang Central People’s Hospital from January 2024 to December 2025. ENAD was defined as Bristol Stool Form Scale type ≥6 plus either ≥3 bowel movements/day or stool output >500 g/24 h after EN initiation. Candidate predictors covered demographic, neurological, nutritional, laboratory, EN-related, medication, and organ-support variables. Missing values were handled within five-fold cross-validation training folds. LASSO logistic regression was used for feature screening, and six algorithms were compared using five-fold cross-validated out-of-fold predictions. Performance was assessed using discrimination, precision-recall performance, calibration-related metrics, confusion-matrix indices, decision-curve analysis, and SHAP interpretation.

**Results:**

Among 374 patients, 105 developed ENAD (28.1%). Patients with ENAD had higher NRS-2002, APACHE II, NIHSS, and mRS scores; lower GCS scores; longer ICU stay and mechanical ventilation; higher EN infusion rates and CRP; and lower albumin. The random forest model showed the best overall internal performance, with AUC 0.969, AUPRC 0.921, sensitivity 0.943, specificity 0.888, negative predictive value 0.98, and F1 score 0.85 at a threshold of 0.45. SHAP identified EN infusion rate, CRP, NRS-2002 score, albumin, mRS score, APACHE II score, and NIHSS score as leading contributors.

**Conclusion:**

Pending external validation, the interpretable random forest model can support an early ENAD risk pathway for ICU patients with ischemic stroke receiving EN. High-risk predictions should trigger structured stool monitoring, feeding-rate and delivered-versus-prescribed EN review, medication review, skin protection, fluid-electrolyte surveillance, and early nutrition-support consultation. Future work should prioritize multicenter validation, calibration updating, parsimonious model comparison, and prospective impact testing before routine clinical deployment.

## 1 Introduction

Ischemic stroke remains a leading cause of neurological disability and frequently requires intensive care when complicated by impaired consciousness, respiratory failure, infection, hemodynamic instability, or severe dysphagia (1, 2). In this context, timely nutritional support is not only a supportive measure but also a component of organ-protective critical care. International and Chinese critical-care nutrition guidelines recommend early enteral nutrition when the gastrointestinal tract is functional and no contraindication exists, because EN helps preserve gut integrity, supports immune and metabolic homeostasis, and facilitates the delivery of protein-energy targets in patients unable to maintain safe oral intake (3–6). Stroke-specific nutritional management is particularly important because dysphagia, aspiration risk, immobility, and neurological disability can prolong tube feeding and increase exposure to ICU interventions (1, 2, 7).

Despite these benefits, EN is frequently limited by gastrointestinal intolerance, among which ENAD is one of the most clinically visible and nursing-intensive complications. Reported diarrhea rates in critically ill patients vary widely because of differences in case mix, stool definitions, observation windows, EN protocols, formula composition, antibiotic exposure, and the intensity of nursing documentation (8–11). Previous studies have linked diarrhea during critical illness or EN to illness severity, antibiotic exposure, hypoalbuminemia, inflammatory status, mechanical ventilation, renal replacement therapy, feeding regimen, and care-related factors (8–13). These risk factors are rarely isolated; rather, they interact across nutritional, inflammatory, neurological, pharmacological, and care-process domains.

ENAD is clinically relevant because it can interrupt nutritional delivery, worsen fluid and electrolyte imbalance, aggravate perianal skin injury, increase the need for stool-management and infection-control measures, and add to nursing workload (8–12, 14, 15). In patients with ischemic stroke, these consequences may be amplified. Neurological impairment may reduce mobility and intestinal motility; dysphagia and reduced consciousness prolong enteral feeding; infection and antibiotics alter the gut microbiota; and critical-illness severity may compromise intestinal perfusion and barrier function. Therefore, early recognition of high-risk patients is central to individualized nutrition monitoring, rational feeding advancement, skin protection, medication review, and interdisciplinary decision-making.

Existing ENAD studies have mainly focused on general ICU populations or conventional regression-based risk-factor analysis (12, 13, 16). Although these studies have clarified important clinical associations, they may not fully capture nonlinear relationships and higher-order interactions among feeding delivery, nutritional reserve, inflammation, neurological severity, and organ-support variables. Machine-learning methods can accommodate complex variable structures and may improve individualized risk stratification when developed and reported transparently (17–21). However, black-box prediction alone is insufficient for bedside use. Clinicians and nurses need interpretable models that identify not only who is at risk, but also which modifiable or monitorable factors contribute to each prediction (19–21).

Disease-specific prediction models for ENAD among neurocritical patients with ischemic stroke remain limited. This gap is important because ischemic stroke patients in the ICU differ from general ICU cohorts in dysphagia burden, neurological disability, autonomic and mobility impairment, and stroke-related care pathways. The present study therefore aimed to develop, compare, and internally validate machine-learning models for predicting ENAD in ICU patients with ischemic stroke receiving EN, and to interpret the final model using SHAP to identify clinically meaningful risk signals for nursing and nutritional management. Available evidence on sociodemographic inequalities in ENAD among ICU stroke patients is limited; because the present dataset included age and sex but not ethnicity or socioeconomic indicators, the model should not be interpreted as evaluating or correcting such inequalities.

## 2 Materials and methods

### 2.1 Study design and reporting

This was a single-center retrospective cohort study using routinely collected clinical data from Yichang Central People’s Hospital. The study was designed as a clinical prediction-model development and internal-validation study and was reported according to the TRIPOD+AI statement for clinical prediction models using regression or machine-learning methods; the completed checklist is provided in Supplementary material 1. The study was conducted in accordance with the Declaration of Helsinki and approved by the Ethics Committee of Yichang Central People’s Hospital (approval No. 2025–394-01). Because the study used retrospective de-identified clinical data, the requirement for written informed consent was waived by the ethics committee. No prospective study protocol was registered or prepared, and patients or members of the public were not involved in study design, conduct, interpretation, or reporting because of the retrospective de-identified design.

### 2.2 Study population

Hospitalized patients with ischemic stroke between January 2024 and December 2025 were screened. Eligible patients were adults with ischemic stroke confirmed by cranial computed tomography or magnetic resonance imaging, admitted to the ICU, and treated with EN during the ICU stay. Patients were excluded if they had no ICU admission record, did not receive EN during the ICU stay, had diarrhea before EN initiation, had duplicate admissions beyond the first eligible record, or had records that could not support reliable outcome or predictor verification after final data-quality review. Study size was determined by including all eligible records during the predefined accrual period; no formal *a priori* sample-size calculation was performed.

**Figure 1:**
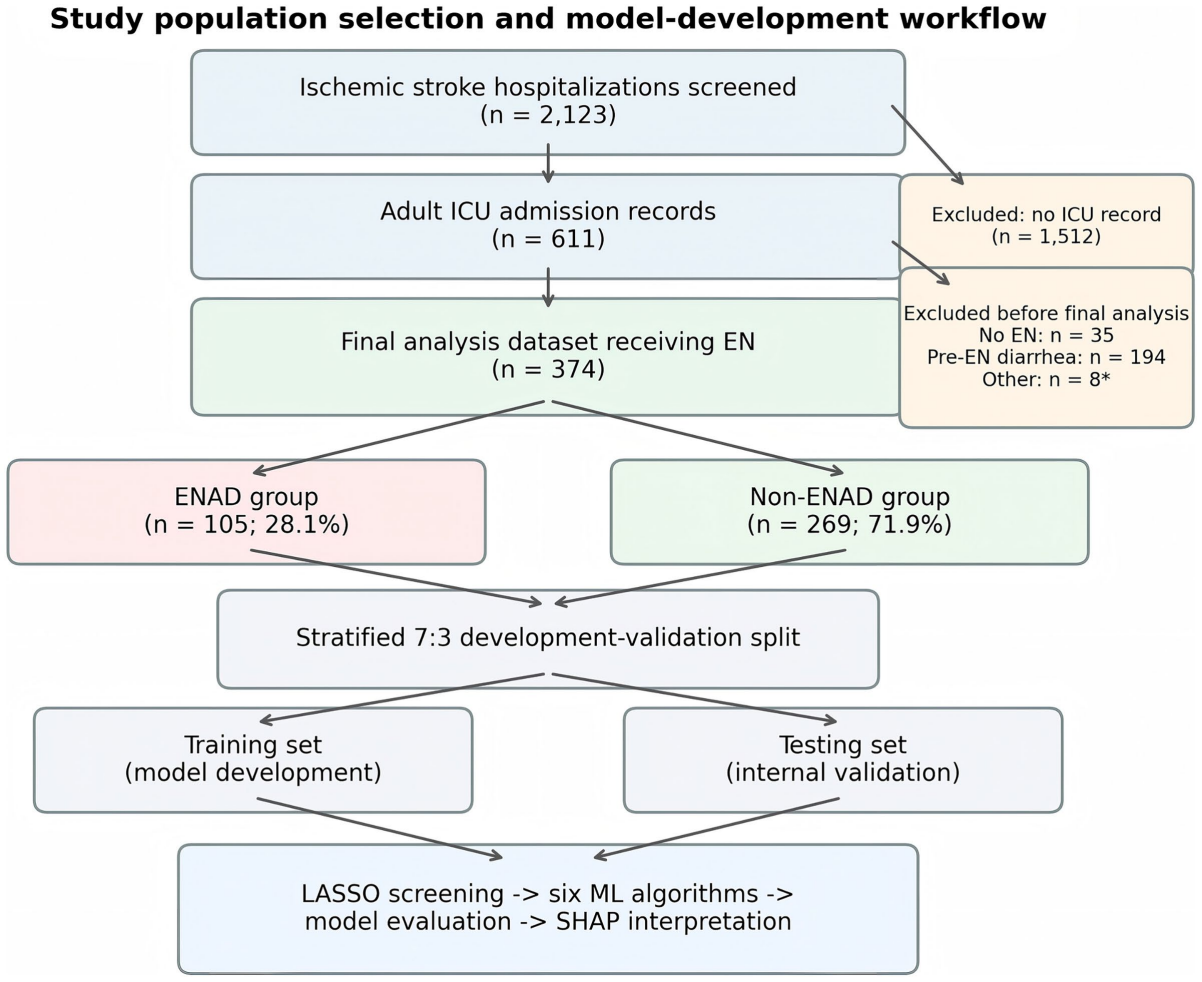
Study population selection and five-fold cross-validated model-development workflow. EN, enteral nutrition; ENAD, enteral nutrition-associated diarrhea; ICU, intensive care unit; LASSO, least absolute shrinkage and selection operator; SHAP, SHapley Additive exPlanations.

The patient-selection process is shown in Figure 1. Of 2,123 screened ischemic stroke hospitalizations, 1,512 without ICU admission records were excluded. Among 611 adult ICU admission records, 35 patients did not receive EN and 194 had pre-EN diarrhea. After final eligibility confirmation, de-duplication, and data-quality review, 374 patients receiving EN were retained in the analytic cohort, including 105 patients with ENAD and 269 without ENAD.

### 2.3 Outcome definition

The primary outcome was ENAD during the ICU stay. Following the operational definition used in recent ENAD prediction research and stool-form criteria for diarrhea assessment, ENAD was defined as new-onset diarrhea after EN initiation with concurrent evidence of abnormal stool consistency and increased stool frequency or volume: Bristol Stool Form Scale type ≥6, plus either ≥3 bowel movements per day or total stool output >500 g/24 h (16, 22). Outcome information was abstracted from routine ICU nursing records and clinical charts. Patients with diarrhea before EN initiation were excluded to reduce outcome misclassification and to strengthen the temporal association with EN exposure.

### 2.4 Candidate predictors and data preprocessing

Candidate predictors were selected according to biological plausibility, availability in routine ICU records, and relevance to EN tolerance. Predictor domains included demographic characteristics, smoking and alcohol status, comorbidities, neurological and functional severity scores, critical-illness severity, vital signs, arterial blood gas variables, inflammatory and nutritional markers, liver and renal function, electrolytes, fluid balance, fasting duration, EN formula type, feeding dose and infusion rate, parenteral nutrition, antibiotic therapy, prokinetic therapy, prophylactic laxative use, sedative and analgesic exposure, vasoactive drugs, oxygen therapy, FiO2, and respiratory support-related variables. The complete candidate-predictor domains and variable-level missingness are reported in Supplementary Tables S1, S2C.

All model-development procedures were embedded within five-fold cross-validation. To avoid information leakage, imputation, scaling when required, feature screening, class-imbalance handling, model fitting, and prediction generation were performed using the training fold only and then applied to the validation fold to obtain out-of-fold predictions. Missing continuous predictors were imputed using the median of the corresponding training fold, and missing categorical predictors were imputed using the most frequent category of the corresponding training fold. Outcome values were not imputed, numeric zero was not treated as missing, and variables considered potential post-outcome or leakage variables were excluded before model development. Continuous variables were checked for distributional normality using the Shapiro–Wilk test. Variables with non-normal distribution in either group were summarized as median (interquartile range) and compared using the Mann–Whitney U test. Approximately normally distributed continuous variables were summarized as mean ± standard deviation and compared using Welch’s t-test. Categorical variables were summarized as frequency (percentage) and compared using Pearson’s chi-square test or Fisher’s exact test, as appropriate. Two-sided *p* < 0.05 was considered statistically significant.

### 2.5 Feature selection and model development

LASSO logistic regression was used for feature screening and shrinkage. The screened variables were entered into six candidate algorithms: random forest, XGBoost, gradient boosting, support vector machine, regularized logistic regression, and extra trees (23–28). The primary internal-validation analysis used five-fold cross-validated out-of-fold predictions for all six algorithms. Hyperparameter specification was applied consistently across algorithms within the cross-validation framework; effective final hyperparameters, implemented candidate search spaces, and internal cross-validation criteria are reported in Supplementary Table S3A. Class imbalance was handled within training folds using class-weighting for applicable algorithms and scale_pos_weight for XGBoost; no oversampling was performed. The final model was selected according to the overall balance of discrimination, positive-class recognition, calibration-related metrics, decision-curve net benefit, and clinically meaningful sensitivity and specificity, rather than a single metric alone.

### 2.6 Model evaluation, interpretation, and sensitivity analyses

Discrimination was quantified using the area under the receiver operating characteristic curve (AUC). Because ENAD events were less frequent than non-events, the area under the precision-recall curve (AUPRC) was also evaluated. Additional metrics included Brier score, accuracy, sensitivity, specificity, positive predictive value, negative predictive value, F1 score, selected probability threshold, and confusion-matrix counts. The primary model output was an individualized predicted probability of ENAD; classification thresholds were selected from the cross-validated predictions to balance sensitivity and specificity while preserving clinical usefulness for nursing risk screening. Calibration curves were used to examine agreement between predicted and observed risk, and decision-curve analysis was used to evaluate potential clinical net benefit across threshold probabilities (29, 30). SHAP analysis was applied to the final random forest model refitted with the same preprocessing and hyperparameter settings to quantify global feature importance and patient-level feature contributions (31). To examine whether correlated variables distorted model interpretation, we performed Spearman correlation analysis, variance inflation factor (VIF) diagnostics, neurological/severity-score principal component analysis (PCA), reduced neurological-score modeling, and bootstrap SHAP-rank stability analysis. To address bedside usability, the full model was compared with a study-specific 12-predictor model and a Liao-compatible overlapping-predictor model. These additional analyses are reported in Supplementary Tables S5–S9 and Supplementary Figures S2–S8. Formal fairness modeling was not performed because the retrospective single-center dataset contained limited sociodemographic information beyond age and sex; this is addressed as a limitation.

## 3 Results

### 3.1 Study population and ENAD incidence

The final analytic cohort included 374 ICU patients with ischemic stroke receiving EN. ENAD occurred in 105 patients, corresponding to an incidence of 28.1%, whereas 269 patients (71.9%) did not develop ENAD. Table 1 presents baseline characteristics according to ENAD status and variable distribution.

**Table 1:** Baseline characteristics according to ENAD status in the final analytic cohort.

Characteristic	All patients (*n* = 374)	Non-ENAD (*n* = 269)	ENAD (*n* = 105)	*P* value
Age, years	69.00 (61.00–75.00)	69.00 (61.00–76.00)	68.00 (59.00–73.00)	0.178
Male sex	238 (63.6%)	172 (63.9%)	66 (62.9%)	0.845
Body mass index, kg/m^2^	23.00 (21.20–25.30)	23.00 (21.00–25.20)	23.20 (22.00–25.40)	0.225
Current or former smoking	117 (31.3%)	87 (32.3%)	30 (28.6%)	0.480
Alcohol drinking	126 (33.7%)	93 (34.6%)	33 (31.4%)	0.563
NRS-2002 score	3.00 (3.00–4.00)	3.00 (3.00–4.00)	5.00 (4.00–5.00)	<0.001
APACHE II score	20.00 (16.00–23.75)	19.00 (14.00–22.00)	23.00 (20.00–26.00)	<0.001
GCS score	7.00 (5.00–10.00)	7.00 (5.00–10.00)	6.00 (4.00–8.00)	<0.001
NIHSS score	13.00 (8.25–17.00)	12.00 (5.00–15.00)	16.00 (13.00–20.00)	<0.001
mRS score	2.00 (1.00–3.00)	2.00 (1.00–3.00)	4.00 (3.00–4.00)	<0.001
ICU length of stay, h	93.25 (62.12–145.38)	78.00 (49.00–112.50)	165.50 (112.50–270.25)	<0.001
Mechanical ventilation time, h	17.25 (0.00–86.75)	0.00 (0.00–63.00)	85.00 (0.00–198.50)	<0.001
Hypertension	260 (69.5%)	188 (69.9%)	72 (68.6%)	0.804
Fasting duration before EN: <=24 h	199 (53.2%)	149 (55.4%)	50 (47.6%)	0.277
24–48 h	122 (32.6%)	86 (32.0%)	36 (34.3%)	
>48 h or not documented	53 (14.2%)	34 (12.6%)	19 (18.1%)	
Peptide-based enteral formula	212 (56.7%)	145 (53.9%)	67 (63.8%)	0.082
Enteral infusion rate, mL/h	37.50 (30.00–50.00)	30.00 (30.00–40.00)	50.00 (40.00–50.00)	<0.001
Antibiotic therapy	229 (61.2%)	156 (58.0%)	73 (69.5%)	0.040
Prokinetic therapy	116 (31.0%)	73 (27.1%)	43 (41.0%)	0.009
Prophylactic laxative use	106 (28.3%)	65 (24.2%)	41 (39.0%)	0.004
Any sedative agent	135 (36.1%)	84 (31.2%)	51 (48.6%)	0.002
Any analgesic agent	179 (47.9%)	109 (40.5%)	70 (66.7%)	<0.001
Daily potassium supplementation dose	0.00 (0.00–3.00)	0.00 (0.00–2.00)	3.00 (0.00–3.00)	<0.001
Mean arterial pressure, mmHg	95.11 ± 12.58	95.69 ± 12.70	93.63 ± 12.19	0.147
pH	7.39 (7.36–7.43)	7.40 (7.35–7.42)	7.39 (7.36–7.43)	0.988
Potassium, mmol/L	3.80 (3.55–4.04)	3.78 (3.55–4.03)	3.86 (3.56–4.10)	0.361
Sodium, mmol/L	137.87 (135.54–140.05)	137.88 (135.80–139.84)	137.86 (134.86–140.41)	0.743
Calcium, mmol/L	1.08 (1.04–1.12)	1.08 (1.04–1.12)	1.09 (1.05–1.12)	0.366
Glucose, mmol/L	7.49 (6.21–9.31)	7.37 (6.14–8.96)	7.70 (6.51–10.63)	0.040
Lactate, mmol/L	1.35 (1.09–1.77)	1.32 (1.08–1.77)	1.38 (1.14–1.80)	0.129
Bicarbonate, mmol/L	23.32 ± 2.79	23.23 ± 2.75	23.56 ± 2.86	0.314
Serum osmolality, mOsm/kg	278.00 (274.00–282.00)	278.00 (274.00–282.00)	279.00 (273.00–283.00)	0.382
Oxygen saturation, %	98.70 (96.93–99.40)	98.70 (97.00–99.40)	98.70 (96.90–99.40)	0.642
PaO2/FiO2 ratio	501.60 (380.87–701.99)	507.90 (394.95–729.36)	467.52 (369.61–665.10)	0.221
White blood cell count, 10^9/L	9.38 (7.48–11.96)	9.12 (7.18–11.77)	10.27 (8.66–13.17)	0.005
Hemoglobin, g/L	125.00 (110.00–139.00)	127.00 (113.00–141.00)	119.00 (100.00–134.00)	<0.001
Platelet count, 10^9/L	184.00 (141.25–221.00)	185.00 (141.00–221.00)	181.00 (143.00–220.00)	0.866
Neutrophil count, 10^9/L	7.57 (5.86–10.24)	7.19 (5.69–9.75)	8.46 (6.51–11.46)	0.006
Lymphocyte count, 10^9/L	0.95 (0.69–1.34)	0.98 (0.76–1.43)	0.84 (0.58–1.13)	<0.001
Urea, mmol/L	5.81 (4.46–7.80)	5.87 (4.39–7.75)	5.79 (4.65–7.80)	0.497
Creatinine, μmol/L	72.00 (57.00–92.50)	71.00 (56.00–91.00)	74.50 (59.00–95.25)	0.450
C-reactive protein, mg/L	18.12 (4.42–61.56)	9.17 (3.58–30.98)	71.96 (35.12–119.77)	<0.001
Albumin, g/L	36.50 (32.90–39.38)	37.92 (34.80–40.50)	32.50 (31.10–34.60)	<0.001
Prothrombin time, s	14.00 (13.40–14.60)	14.00 (13.40–14.60)	13.90 (13.47–14.60)	0.980
FiO2, %	33.00 (33.00–40.00)	33.00 (33.00–40.00)	40.00 (33.00–45.00)	<0.001

Values are presented as mean ± standard deviation, median (interquartile range), or *n* (%), as appropriate. APACHE II, acute physiology and chronic health evaluation II; EN, enteral nutrition; ENAD, enteral nutrition-associated diarrhea; FiO2, fraction of inspired oxygen; GCS, Glasgow Coma Scale; ICU, intensive care unit; mRS, modified Rankin Scale; NIHSS, National Institutes of Health Stroke Scale; NRS-2002, nutritional risk screening 2002.

Patients who developed ENAD had a more severe nutritional-neurological profile than those without ENAD. The ENAD group had a higher NRS-2002 score [5.00 (4.00–5.00) vs. 3.00 (3.00–4.00), *p* < 0.001], APACHE II score [23.00 (20.00–26.00) vs. 19.00 (14.00–22.00), *p* < 0.001], NIHSS score [16.00 (13.00–20.00) vs. 12.00 [5.00–15.00], *p* < 0.001], and mRS score [4.00 (3.00–4.00) vs. 2.00 (1.00–3.00), *p* < 0.001], and a lower GCS score [6.00 (4.00–8.00) vs. 7.00 (5.00–10.00), *p* < 0.001]. ICU length of stay and mechanical ventilation time were also longer in the ENAD group. Feeding and treatment-related differences included a higher enteral infusion rate, more frequent antibiotic therapy, prokinetic therapy, prophylactic laxative use, sedative exposure, and analgesic exposure. Laboratory differences were most prominent for CRP and albumin: CRP was markedly higher in the ENAD group [71.96 (35.12–119.77) vs. 9.17 (3.58–30.98) mg/L, *p* < 0.001], whereas albumin was lower [32.50 (31.10–34.60) vs. 37.92 (34.80–40.50) g/L, *p* < 0.001].

### 3.2 Predictor selection

LASSO-based feature screening identified a compact but clinically multidimensional predictor set. Ranked by aggregated native-model importance after shrinkage, the leading predictors were enteral infusion rate, albumin, mRS score, NRS-2002 score, CRP, APACHE II score, NIHSS score, hemoglobin, daily potassium dose, GCS score, coefficient of variation, FiO2, lactate, PaO2/FiO2 ratio, lymphocyte count, total hemoglobin, glucose, calcium, SaO2, and white blood cell count (Supplementary Table S4). Hemoglobin and total hemoglobin were retained as separate variables because they represented different source measurements: routine blood-count hemoglobin and blood-gas/co-oximetry total hemoglobin, respectively. A near-duplicate assessment after scaling tHb by 10 showed incomplete agreement; both variables had acceptable VIFs and were therefore retained with collinearity diagnostics (Supplementary Tables S5, S6). These variables captured nutrition delivery, nutritional reserve, inflammation, neurological disability, critical-illness severity, respiratory status, and metabolic status.

### 3.3 Comparative performance of machine-learning models

**Figure 2:**
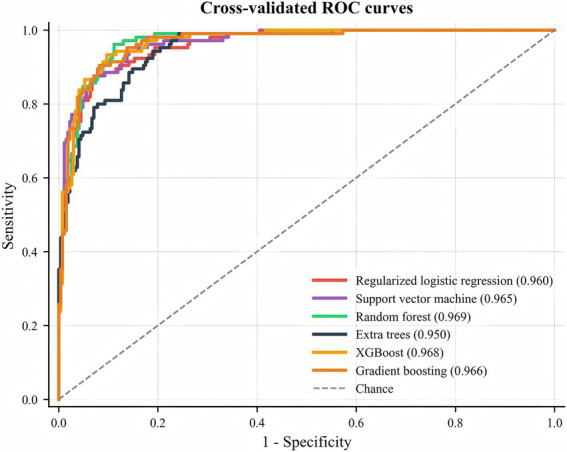
Cross-validated receiver operating characteristic curves for six candidate machine-learning models. AUC values are displayed in parentheses in the figure legend.

Across the six candidate models, five-fold cross-validated out-of-fold performance was consistently high (Table 2; Figure 2; Supplementary Table S3B). The random forest model achieved the highest AUC (0.969; 95% CI, 0.95–0.98), marginally exceeding XGBoost (0.968), gradient boosting (0.966), support vector machine (0.965), regularized logistic regression (0.960), and extra trees (0.950). The random forest also showed strong positive-class recognition with an AUPRC of 0.921 (95% CI, 0.88–0.96), slightly below support vector machine (0.924) but comparable to XGBoost and gradient boosting (both 0.919).

**Table 2:** Five-fold cross-validated internal performance of candidate machine-learning models.

**Model**	**AUC (95% CI)**	**AUPRC (95% CI)**	**Brier (95% CI)**	**Accuracy**	**Sensitivity**	**Specificity**	**F1**
Random forest	0.969 (0.95–0.98)	0.921 (0.88–0.96)	0.12 (0.11–0.13)	0.904	0.943	0.888	0.85
XGBoost	0.968 (0.95–0.98)	0.919 (0.87–0.96)	0.062 (0.05–0.08)	0.91	0.93	0.90	0.85
Gradient boosting	0.966 (0.95–0.98)	0.919 (0.87–0.96)	0.07 (0.05–0.09)	0.89	0.92	0.87	0.82
Support vector machine	0.965 (0.95–0.98)	0.924 (0.88–0.96)	0.06 (0.05–0.08)	0.90	0.89	0.91	0.83
Regularized logistic regression	0.960 (0.94–0.98)	0.912 (0.86–0.96)	0.07 (0.05–0.10)	0.87	0.92	0.85	0.80
Extra trees	0.950 (0.93–0.97)	0.891 (0.84–0.94)	0.16 (0.15–0.17)	0.84	0.95	0.80	0.77

Performance estimates are based on five-fold cross-validated out-of-fold predictions. AUC, area under the receiver operating characteristic curve; AUPRC, area under the precision-recall curve; CI, confidence interval.

At a probability threshold of 0.45, the random forest model achieved accuracy of 0.904, sensitivity of 0.943, specificity of 0.888, PPV of 0.77, NPV of 0.98, and F1 score of 0.85. XGBoost showed the lowest Brier score (0.062) and favorable probability calibration, whereas random forest provided the best overall combination of discrimination, sensitivity, specificity, and decision-curve performance. Extra trees showed the lowest discrimination and specificity among the candidate models. Full confusion-matrix counts and 95% CIs for all models are provided in Supplementary Table S3B.

### 3.4 Calibration, decision-curve performance, and final model selection

**Figure 3:**
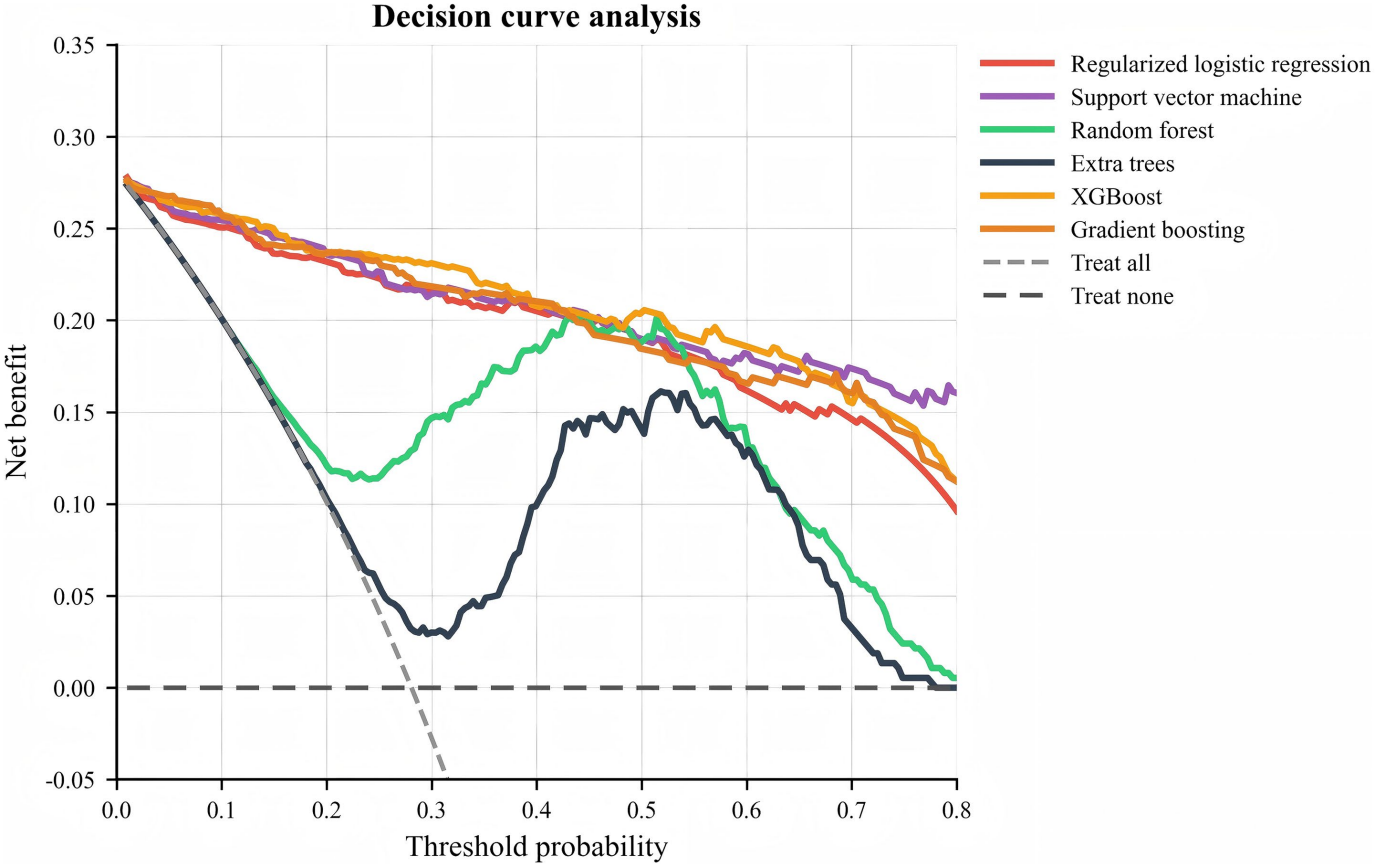
Decision-curve analysis for six candidate machine-learning models. Net benefit is shown across threshold probabilities from 0 to 0.80. The dashed lines represent treat-all and treat-none strategies.

Decision-curve analysis based on cross-validated predictions demonstrated that random forest, XGBoost, and gradient boosting achieved higher clinical net benefit than treat-all or treat-none strategies across a broad threshold range of approximately 0.10–0.80 (Figure 3). The random forest model maintained a wider and more stable net-benefit profile than the other models, supporting its selection as the final model. Calibration analysis indicated that XGBoost and gradient boosting showed the most favorable probability calibration, with random forest performing slightly less well but remaining clinically competitive when considered alongside discrimination and net benefit.

### 3.5 SHAP-based interpretation of the final random forest model

**Figure 4:**
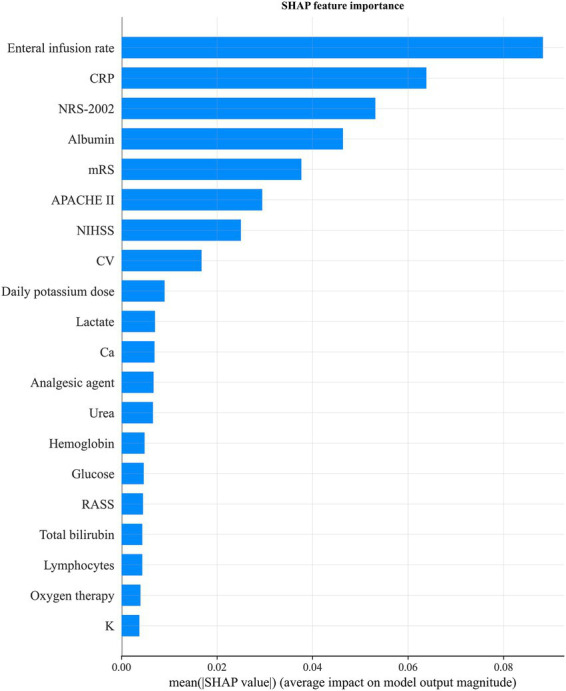
SHAP-based interpretation of the final random forest model. **(A)** Mean absolute SHAP values showing global feature importance. **(B)** SHAP beeswarm plot showing the direction and magnitude of feature effects. Red points indicate higher feature values and blue points indicate lower feature values.

SHAP interpretation revealed that enteral infusion rate, CRP, NRS-2002 score, albumin, and mRS score were the top contributors to random forest predictions (Figure 4). Additional contributors included APACHE II score, NIHSS score, coefficient of variation, daily potassium dose, lactate, calcium, analgesic agent, urea, hemoglobin, glucose, Richmond Agitation-Sedation Scale score, total bilirubin, lymphocyte count, oxygen therapy, and potassium.

**Figure 5:**
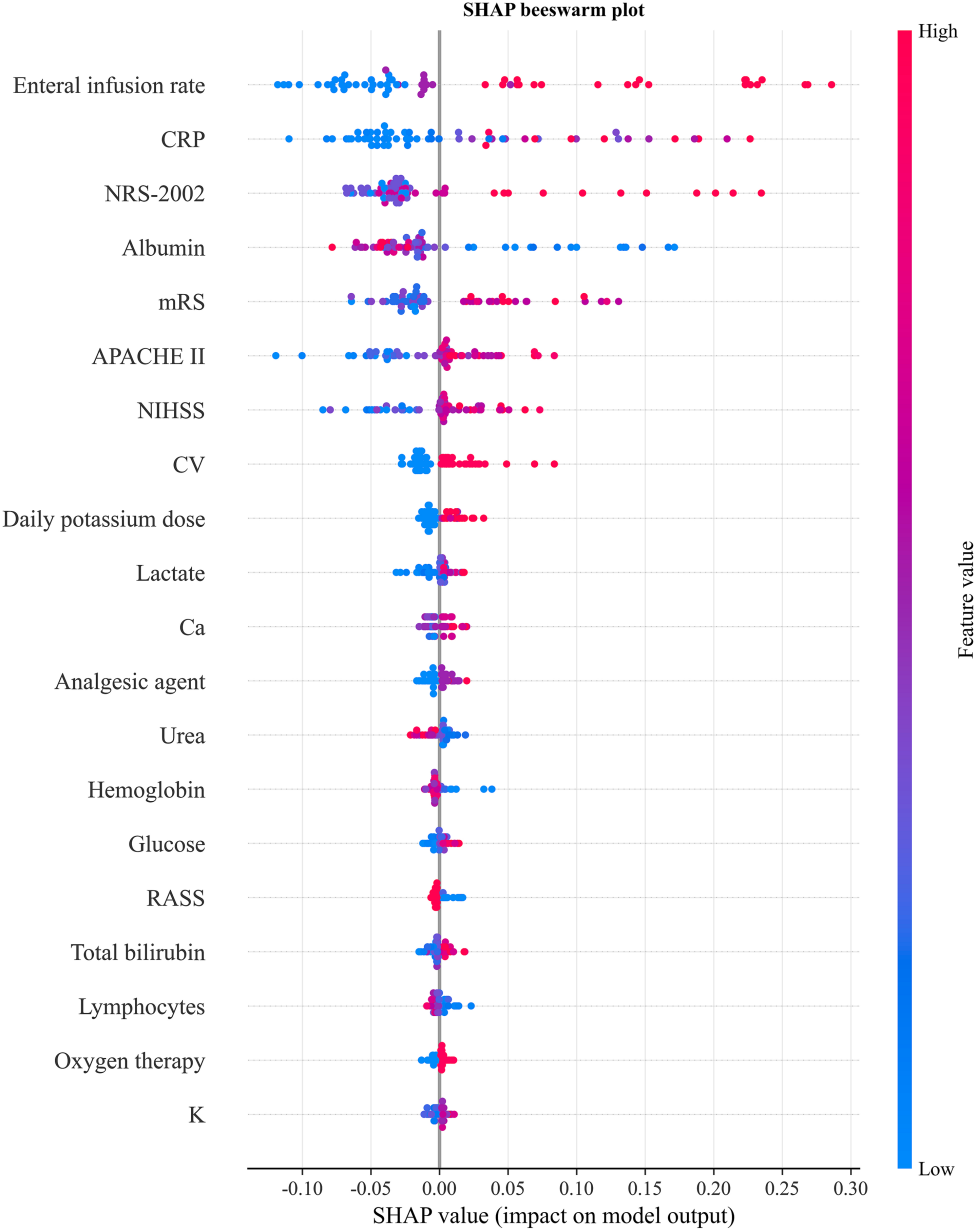
SHAP beeswarm plot of the final random forest model. Each dot represents one patient for the corresponding predictor. The x-axis shows the SHAP value, indicating the direction and magnitude of each feature’s contribution to the predicted probability of enteral nutrition-associated diarrhea. Red points indicate higher feature values and blue points indicate lower feature values. Positive SHAP values increase the predicted risk of enteral nutrition-associated diarrhea, whereas negative SHAP values decrease the predicted risk.

The SHAP beeswarm plot showed clinically plausible directions of effect. Higher enteral infusion rate, CRP, NRS-2002 score, and mRS score tended to push predictions toward higher ENAD risk, whereas higher albumin tended to push predictions toward lower risk. A representative waterfall plot further demonstrated how an individual prediction could be driven upward by high enteral infusion rate, NRS-2002 score, albumin-related contribution, CRP, mRS score, coefficient of variation, and APACHE II score. These findings suggest that ENAD prediction was driven by an interpretable combination of nutrition delivery intensity, inflammatory burden, nutritional risk, neurological disability, and critical-illness severity (Figure 5).

### 3.6 Collinearity, SHAP-rank stability, and parsimonious-model comparisons

VIFs for the selected final-model predictors were all below 5, including hemoglobin (3.84) and total hemoglobin (3.68), indicating no serious collinearity among the main predictors (Supplementary Table S6; Supplementary Figure S4). Replacing the neurological/severity-score cluster with a first principal component or using a reduced neurological-score set produced similar discrimination to the primary full random forest model, suggesting that redundant neurological scoring did not materially drive the model signal (Supplementary Tables S7A–S7C; Supplementary Figures S5, S6). Bootstrap SHAP analysis also showed stable ranking of the leading contributors, with CRP, albumin, enteral infusion rate, mRS score, NRS-2002 score, APACHE II score, and NIHSS score appearing consistently among top-ranked predictors (Supplementary Table S8; Supplementary Figure S7).

For clinical usability, the full random forest model was compared with a study-specific 12-predictor random forest model, a Liao-compatible overlapping-predictor model, and a 12-predictor regularized logistic-regression model. The study-specific 12-predictor random forest model showed discrimination similar to the full model, whereas the Liao-compatible model performed less well because several Liao predictors were unavailable in the present cohort. The 12-predictor logistic model also showed high discrimination. These findings support further external validation of parsimonious models that may be easier to implement at the bedside (Supplementary Tables S9A–S9B, Supplementary Figure S8).

## 4 Discussion

### 4.1 Principal findings

In this single-center cohort of ICU patients with ischemic stroke receiving EN, ENAD occurred in 28.1% of patients. The random forest model achieved the strongest overall internal predictive performance among six candidate algorithms using five-fold cross-validated out-of-fold predictions. Importantly, its clinical value was not limited to a high AUC. The model also showed strong AUPRC, high sensitivity, acceptable specificity, high negative predictive value, and favorable decision-curve net benefit, which are particularly relevant for an ICU nursing-risk problem where missed high-risk patients may result in delayed feeding adjustment, inadequate stool monitoring, electrolyte disturbance, skin injury, and interruption of nutritional support (8, 9, 12, 15).

The model interpretation is clinically coherent. The leading SHAP predictors were not isolated statistical artifacts, but variables that represent core pathophysiological and care-process domains: feeding delivery intensity, inflammatory burden, nutritional risk and reserve, neurological disability, and critical-illness severity. This multidimensional pattern is consistent with current understanding that ENAD in critical illness is driven by the interaction of host vulnerability, gastrointestinal barrier dysfunction, microbiota disruption, medication exposure, and EN administration processes rather than by formula administration alone (8–16).

### 4.2 Incidence and baseline phenotype of ENAD in ICU ischemic stroke patients

The observed ENAD incidence was within the broad range reported in ICU studies, where diarrhea estimates vary substantially according to stool criteria, patient population, and documentation intensity (8–11). Compared with the general ICU machine-learning study by Liao et al., which reported a diarrhea incidence of 28.7% in ICU patients receiving EN, the incidence in our ischemic stroke ICU cohort was similar, supporting the clinical relevance of ENAD across critical-care settings (16). However, the baseline phenotype in the present study was more neurocritical-care specific. ENAD patients had significantly higher NIHSS and mRS scores and lower GCS scores, suggesting that stroke severity, disability, and impaired consciousness may be important contextual drivers of feeding intolerance.

These neurological differences have practical implications. Severe stroke can impair swallowing, consciousness, cough reflex, mobility, and autonomic regulation. These factors may prolong tube feeding, increase the need for mechanical ventilation or sedation, delay mobilization, and complicate bowel management. In our data, ICU length of stay and mechanical ventilation time were both markedly longer among patients with ENAD. Although causality cannot be inferred from a retrospective cohort, the pattern suggests that ENAD may be part of a broader high-acuity trajectory rather than a standalone gastrointestinal event. This interpretation is consistent with previous critical-care data showing that diarrhea during critical illness is associated with greater illness burden and more intensive treatment exposure (8, 9, 12).

### 4.3 Feeding delivery: enteral infusion rate as a modifiable risk signal

Enteral infusion rate was the most influential SHAP predictor and differed significantly between ENAD and non-ENAD groups. Higher rates may exceed absorptive or motility capacity in neurologically and physiologically vulnerable patients, particularly when critical illness is accompanied by hypoperfusion, inflammation, sedation, antibiotic exposure, or delayed gastric emptying. This does not imply that feeds should be reduced indiscriminately, because underfeeding is also harmful in critical illness (3–6, 15). Rather, the finding supports individualized feeding advancement and closer tolerance monitoring among high-risk patients.

This result aligns with bedside nutrition principles emphasizing gradual progression, evaluation of tolerance, and response to gastrointestinal symptoms rather than rigid rate escalation (4, 5, 15). The clinical contribution of the model is that it can identify patients in whom a given infusion rate may represent excessive physiological stress. For high-risk patients, practical nursing actions may include more frequent stool assessment, review of feeding pump settings, verification of prescribed versus delivered EN, evaluation of formula changes, documentation of abdominal symptoms, and early communication with the nutrition-support team.

### 4.4 Inflammatory-nutritional axis: CRP, albumin, and NRS-2002

CRP, albumin, and NRS-2002 were central predictors, indicating that ENAD risk is strongly linked to systemic inflammation and nutritional vulnerability. CRP was markedly higher in the ENAD group and ranked second in SHAP importance. Inflammation may increase intestinal permeability, alter absorptive function, impair microcirculation, and contribute to dysbiosis, thereby lowering gastrointestinal tolerance during EN (12, 32). Conversely, albumin was lower in the ENAD group and showed a protective direction in SHAP analysis when values were higher. Hypoalbuminemia may reflect malnutrition, inflammation, capillary leak, reduced oncotic pressure, and diminished physiological reserve, all of which can predispose patients to fluid shifts and gastrointestinal dysfunction (13, 32, 33).

The strong contribution of NRS-2002 is also clinically meaningful. NRS-2002 integrates nutritional status with disease severity, and its high ranking suggests that ENAD prediction should not be separated from overall nutritional-risk assessment (33). In nursing practice, a patient with high NRS-2002, elevated CRP, and low albumin should be viewed as having both high need for nutrition and reduced tolerance reserve. This paradox emphasizes the need for individualized delivery rather than simple escalation or interruption. The model may support a balanced strategy: maintain nutritional goals while intensifying tolerance surveillance, electrolyte monitoring, skin protection, and early multidisciplinary review.

### 4.5 Neurological and critical-illness severity as amplifiers of ENAD risk

Neurological severity and critical-illness severity jointly contributed to ENAD predictions. NIHSS, mRS, GCS, and APACHE II were all prominent in baseline comparisons and model interpretation. Higher NIHSS and mRS scores may indicate severe neurological deficit, immobility, dysphagia, and higher dependence on nursing care. Lower GCS may reflect impaired consciousness and greater need for airway protection, sedation, or mechanical ventilation. APACHE II captures systemic physiological derangement and may reflect the broader biological stress that reduces gastrointestinal reserve.

The association of sedative and analgesic exposure with ENAD in baseline comparisons provides another mechanistic bridge. Sedation and analgesia may alter gastrointestinal motility, delay mobilization, and increase the complexity of feeding management, while analgesic and sedative needs may also be markers of disease severity. Similarly, antibiotic therapy, prokinetic therapy, and prophylactic laxative use differed between groups. These variables should be interpreted cautiously because they may indicate either causal exposure, treatment response to early intolerance, or confounding by severity. Nevertheless, their presence reinforces the value of medication review as part of ENAD prevention and management (8, 9, 12, 13).

### 4.6 Model performance, interpretability, and clinical implementation

Random forest was selected as the final model because it provided the best overall balance across discrimination, positive-class recognition, and clinical utility. Tree-based ensemble methods can capture nonlinear relationships and interactions without requiring strict parametric assumptions (26–28). In the present setting, this flexibility is important because ENAD risk depends on interactions among feeding intensity, inflammatory state, nutritional reserve, neurological disability, and treatment exposures. The supplementary parsimonious-model analyses further suggest that a smaller predictor set may preserve discrimination and improve bedside usability, but such simplified models should be externally validated before replacing the full model.

SHAP analysis improved clinical interpretability by translating the final model into global and patient-level feature contributions (31). For bedside nursing use, interpretability is not a cosmetic addition; it determines whether an alert can be acted upon. A high-risk prediction driven mainly by rapid feeding escalation and high CRP may prompt feeding-rate reassessment and inflammation-focused monitoring, whereas a prediction driven by low albumin, high NRS-2002, and severe neurological disability may prompt nutritional consultation, more cautious advancement, and intensified skin and fluid-electrolyte surveillance. Thus, the model is best conceptualized as an adjunct to structured nursing risk assessment, not as a replacement for clinical judgment.

Before implementation, several steps are required. First, the model should be externally validated in independent neurocritical and general ICU cohorts. Second, calibration should be assessed and updated when applied to institutions with different feeding protocols, documentation practices, formula availability, and patient case mix (20, 21, 23). Third, implementation studies should evaluate whether model-guided nursing interventions reduce ENAD incidence, nutritional interruptions, electrolyte disorders, perianal skin injury, and nursing workload without causing underfeeding. Fourth, electronic-health-record integration should be accompanied by workflow design, alert-threshold testing, staff training, and prospective monitoring for alert fatigue or unintended care changes (19–21, 29). Future external validation and implementation studies should also consider prior work on enteral-feeding intolerance prediction and rapid enteral nutrition administration in acute stroke patients when selecting parsimonious variables and practical alert thresholds (34, 35).

### 4.7 Strengths and limitations

This study has several strengths. It focuses on a clinically important but under-studied neurocritical-care population; incorporates nutritional, neurological, inflammatory, laboratory, medication, and organ-support variables; compares multiple algorithms; evaluates performance beyond AUC, including AUPRC and decision-curve net benefit; reports missingness and hyperparameters in the Supplementary material; and uses SHAP, VIF/PCA sensitivity analyses, and parsimonious-model comparisons to improve interpretability and reproducibility.

Several limitations should be acknowledged. First, this was a single-center retrospective study, and residual confounding, selection bias, and documentation bias are unavoidable. Second, the outcome definition was operational and depended on recorded stool characteristics, frequency, or output; undocumented infectious, medication-related, or formula-related causes may have contributed. Third, only internal five-fold cross-validation was performed, and the number of ENAD events was modest relative to the complexity of machine-learning development; transportable performance may therefore be lower than the internal estimates. Fourth, although imputation was performed within cross-validation folds, variables with moderate or high missingness may still introduce uncertainty. Fifth, the retrospective dataset contained limited sociodemographic information beyond age and sex, so formal fairness analyses across socioeconomic, ethnic, or other demographic groups could not be performed. Sixth, some predictors may have been measured during evolving clinical deterioration, making temporal interpretation difficult. Finally, the clinical impact of model-guided workflow integration remains untested and should be evaluated prospectively before routine implementation.

## 5 Conclusion

In ICU patients with ischemic stroke receiving EN, ENAD risk stratification should be integrated into early nutrition and nursing workflows rather than used as an isolated prediction score. Pending external validation, high-risk predictions can support a structured bedside pathway that includes closer stool surveillance, review of feeding-pump rate and delivered-versus-prescribed EN, medication review for antibiotics, prokinetics, laxatives, sedatives, and analgesics, intensified perianal skin protection, fluid-electrolyte monitoring, and early nutrition-support consultation. Future research should prioritize multicenter external validation, calibration updating across institutions with different EN protocols, comparison of full and parsimonious models, and prospective impact trials testing whether model-guided interventions reduce ENAD, nutrition interruption, electrolyte disturbance, skin injury, and nursing burden without increasing underfeeding.

## Data Availability

The datasets analyzed in this study are not publicly available because they were derived from retrospective hospital electronic medical records and are subject to institutional data-protection and ethics requirements. De-identified data supporting the findings may be made available by the corresponding author upon reasonable request and with approval from Yichang Central People’s Hospital, where applicable. Requests to access these datasets should be directed to JL, 1103179830@qq.com.
