# Supplementary material for: Development and validation of an interpretable machine-learning model for enteral nutrition-associated diarrhea in critically ill patients with ischemic stroke: a retrospective cohort study
Source: Front Nutr. 2026 Jul 14;13:1890164. doi: 10.3389/fnut.2026.1890164 (PMC13407193; doi:10.3389/fnut.2026.1890164)

***Supplementary Material***

**Development and validation of an interpretable machine-learning model for enteral nutrition-associated diarrhea in critically ill patients with ischemic stroke: a retrospective cohort study**

Yang He^1^, Dan Jin^1^, Ming Liu^1^, Jinglan Liu^2*^

**1. TRIPOD+AI reporting checklist**

The completed TRIPOD+AI checklist below reports where each item is addressed in the revised manuscript and supplementary material. Page numbers refer to the rendered revised manuscript submitted with this revision.

| **Section/Topic** | **Item** | **D/E** | **Checklist item** | **Reported in section/table** |
| --- | --- | --- | --- | --- |
| **TITLE** | | | | |
| Title | 1 | D;E | Identify the study as developing or evaluating the performance of a multivariable prediction model, the target population, and the outcome to be predicted | Title |
| **ABSTRACT** | | | | |
| Abstract | 2 | D;E | See TRIPOD+AI for Abstracts checklist | Abstract |
| **INTRODUCTION** | | | | |
| Background | 3a | D;E | Explain the healthcare context (including whether diagnostic or prognostic) and rationale for developing or evaluating the prediction model, including references to existing models | Introduction |
| Background | 3b | D;E | Describe the target population and the intended purpose of the prediction model in the context of the care pathway, including its intended users (e.g., healthcare professionals, patients, public) | Introduction; Discussion: Principal findings; Discussion: Model performance, interpretability, and clinical implementation |
| Background | 3c | D;E | Describe any known health inequalities between sociodemographic groups | Introduction; Discussion: Strengths and limitations |
| Objectives | 4 | D;E | Specify the study objectives, including whether the study describes the development or validation of a prediction model (or both) | Introduction |
| **METHODS** | | | | |
| Data | 5a | D;E | Describe the sources of data separately for the development and evaluation datasets (e.g., randomised trial, cohort, routine care or registry data), the rationale for using these data, and representativeness of the data | Materials and methods: Study design and reporting |
| Data | 5b | D;E | Specify the dates of the collected participant data, including start and end of participant accrual; and, if applicable, end of follow-up | Materials and methods: Study population |
| Participants | 6a | D;E | Specify key elements of the study setting (e.g., primary care, secondary care, general population) including the number and location of centres | Materials and methods: Study design and reporting; Materials and methods: Study population |
| Participants | 6b | D;E | Describe the eligibility criteria for study participants | Materials and methods: Study population |
| Participants | 6c | D;E | Give details of any treatments received, and how they were handled during model development or evaluation, if relevant | Materials and methods: Study population; Materials and methods: Candidate predictors and data preprocessing; Table 1 |
| Data preparation | 7 | D;E | Describe any data pre-processing and quality checking, including whether this was similar across relevant sociodemographic groups | Materials and methods: Candidate predictors and data preprocessing; Supplementary Tables S2B-S2C |
| Outcome | 8a | D;E | Clearly define the outcome that is being predicted and the time horizon, including how and when assessed, the rationale for choosing this outcome, and whether the method of outcome assessment is consistent across sociodemographic groups | Materials and methods: Outcome definition |
| Outcome | 8b | D;E | If outcome assessment requires subjective interpretation, describe the qualifications and demographic characteristics of the outcome assessors | Materials and methods: Outcome definition |
| Outcome | 8c | D;E | Report any actions to blind assessment of the outcome to be predicted | Not applicable; Materials and methods: Outcome definition |
| Predictors | 9a | D | Describe the choice of initial predictors (e.g., literature, previous models, all available predictors) and any pre-selection of predictors before model building | Materials and methods: Candidate predictors and data preprocessing; Supplementary Table S1 |
| Predictors | 9b | D;E | Clearly define all predictors, including how and when they were measured (and any actions to blind assessment of predictors for the outcome and other predictors) | Materials and methods: Candidate predictors and data preprocessing; Supplementary Tables S1-S2C |
| Predictors | 9c | D;E | If predictor measurement requires subjective interpretation, describe the qualifications and demographic characteristics of the predictor assessors | Not applicable; Materials and methods: Candidate predictors and data preprocessing |
| Sample size | 10 | D;E | Explain how the study size was arrived at (separately for development and evaluation), and justify that the study size was sufficient to answer the research question. Include details of any sample size calculation | Materials and methods: Study population |
| Missing data | 11 | D;E | Describe how missing data were handled. Provide reasons for omitting any data | Materials and methods: Candidate predictors and data preprocessing; Supplementary Tables S2B-S2C |
| Analytical methods | 12a | D | Describe how the data were used (e.g., for development and evaluation of model performance) in the analysis, including whether the data were partitioned, considering any sample size requirements | Materials and methods: Feature selection and model development; Table 2 |
| Analytical methods | 12b | D | Depending on the type of model, describe how predictors were handled in the analyses (functional form, rescaling, transformation, or any standardisation). | Materials and methods: Candidate predictors and data preprocessing; Materials and methods: Feature selection and model development |
| Analytical methods | 12c | D | Specify the type of model, rationale, all model-building steps, including any hyperparameter tuning, and method for internal validation | Materials and methods: Feature selection and model development; Supplementary Table S3A |
| Analytical methods | 12d | D;E | Describe if and how any heterogeneity in estimates of model parameter values and model performance was handled and quantified across clusters (e.g., hospitals, countries). See TRIPOD-Cluster for additional considerations | Not applicable; Materials and methods: Study design and reporting; Discussion: Strengths and limitations |
| Analytical methods | 12e | D;E | Specify all measures and plots used (and their rationale) to evaluate model performance (e.g., discrimination, calibration, clinical utility) and, if relevant, to compare multiple models | Materials and methods: Model evaluation, interpretation, and sensitivity analyses; Results: Comparative performance of machine-learning models; Results: Calibration, decision-curve performance, and final model selection; Table 2 |
| Analytical methods | 12f | E | Describe any model updating (e.g., recalibration) arising from the model evaluation, either overall or for particular sociodemographic groups or settings | No model updating; Discussion: Model performance, interpretability, and clinical implementation; Conclusion |
| Analytical methods | 12g | E | For model evaluation, describe how the model predictions were calculated (e.g., formula, code, object, application programming interface) | Materials and methods: Model evaluation, interpretation, and sensitivity analyses; Data availability statement; Supplementary Table S3A |
| Class imbalance | 13 | D;E | If class imbalance methods were used, state why and how this was done, and any subsequent methods to recalibrate the model or the model predictions | Materials and methods: Feature selection and model development; Supplementary Table S3A |
| Fairness | 14 | D;E | Describe any approaches that were used to address model fairness and their rationale | Materials and methods: Model evaluation, interpretation, and sensitivity analyses; Discussion: Strengths and limitations |
| Model output | 15 | D | Specify the output of the prediction model (e.g., probabilities, classification). Provide details and rationale for any classification and how the thresholds were identified | Materials and methods: Model evaluation, interpretation, and sensitivity analyses |
| Training versus evaluation | 16 | D;E | Identify any differences between the development and evaluation data in healthcare setting, eligibility criteria, outcome, and predictors | Materials and methods: Study design and reporting; Materials and methods: Feature selection and model development; internal five-fold cross-validation |
| Ethical approval | 17 | D;E | Name the institutional research board or ethics committee that approved the study and describe the participant-informed consent or the ethics committee waiver of informed consent | Materials and methods: Study design and reporting; Ethics statement |
| **OPEN SCIENCE** | | | | |
| Funding | 18a | D;E | Give the source of funding and the role of the funders for the present study | Funding |
| Conflicts of interest | 18b | D;E | Declare any conflicts of interest and financial disclosures for all authors | Conflict of interest |
| Protocol | 18c | D;E | Indicate where the study protocol can be accessed or state that a protocol was not prepared | Materials and methods: Study design and reporting |
| Registration | 18d | D;E | Provide registration information for the study, including register name and registration number, or state that the study was not registered | Clinical trial registration |
| Data sharing | 18e | D;E | Provide details of the availability of the study data | Data availability statement |
| Code sharing | 18f | D;E | Provide details of the availability of the analytical code | Data availability statement |
| **PATIENT & PUBLIC INVOLVEMENT** | | | | |
| Patient & Public Involvement | 19 | D;E | Provide details of any patient and public involvement during the design, conduct, reporting, interpretation, or dissemination of the study or state no involvement. | Materials and methods: Study design and reporting |
| **RESULTS** | | | | |
| Participants | 20a | D;E | Describe the flow of participants through the study, including the number of participants with and without the outcome and, if applicable, a summary of the follow-up time. A diagram may be helpful. | Results: Study population and ENAD incidence; Figure 1 |
| Participants | 20b | D;E | Report the characteristics overall and, where applicable, for each data source or setting, including the key dates, key predictors (including demographics), treatments received, sample size, number of outcome events, follow-up time, and amount of missing data. A table may be helpful. Report any differences across key demographic groups. | Results: Study population and ENAD incidence; Table 1; Supplementary Tables S2B-S2C |
| Participants | 20c | E | For model evaluation, show a comparison with the development data of the distribution of important predictors (demographics, predictors, and outcome). | Not applicable; no separate external evaluation dataset; Materials and methods: Feature selection and model development |
| Model development | 21 | D;E | Specify the number of participants and outcome events in each analysis (e.g., for model development, hyperparameter tuning, model evaluation) | Results: Study population and ENAD incidence; Results: Comparative performance of machine-learning models; Supplementary Table S3B |
| Model specification | 22 | D | Provide details of the full prediction model (e.g., formula, code, object, application programming interface) to allow predictions in new individuals and to enable third-party evaluation and implementation, including any restrictions to access or re-use (e.g., freely available, proprietary) | Materials and methods: Feature selection and model development; Materials and methods: Model evaluation, interpretation, and sensitivity analyses; Data availability statement; Supplementary Table S3A |
| Model performance | 23a | D;E | Report model performance estimates with confidence intervals, including for any key subgroups (e.g., sociodemographic). Consider plots to aid presentation. | Results: Comparative performance of machine-learning models; Results: Calibration, decision-curve performance, and final model selection; Table 2; Supplementary Table S3B |
| Model performance | 23b | D;E | If examined, report results of any heterogeneity in model performance across clusters. See TRIPOD Cluster for additional details. | Not examined; single-center cohort; Discussion: Strengths and limitations |
| Model updating | 24 | E | Report the results from any model updating, including the updated model and subsequent performance | No model updating; Discussion: Model performance, interpretability, and clinical implementation; Conclusion |

D = items relevant only to the development of a prediction model; E = items relating solely to the evaluation of a prediction model; D;E = items applicable to both development and evaluation. From: Collins GS, Moons KGM, Dhiman P, et al. BMJ 2024;385:e078378. doi:10.1136/bmj-2023-078378.

**2. Supplementary methods**

**2.1 Candidate predictor domains**

Candidate predictors were grouped into clinical domains according to routine ICU availability and biological plausibility for enteral nutrition-associated diarrhea (ENAD).

**Supplementary Table S1. Candidate predictor domains used for model development.**

| **Domain** | **Variables** |
| --- | --- |
| Demographics and lifestyle | Age, sex, body mass index, smoking status, alcohol-drinker status |
| Comorbidities | Hypertension, diabetes, coronary heart disease, hyperlipaemia, recurrent stroke |
| Neurological and critical-illness severity | APACHE II, GCS, NIHSS, mRS, NRS-2002, RASS |
| Vital signs and blood gas | Temperature, heart rate/pulse, respiratory rate, mean arterial pressure, pH, PaO2/FiO2 ratio, FiO2, SaO2, lactate, base excess, bicarbonate |
| Laboratory tests | Albumin, CRP, hemoglobin, total hemoglobin, white blood cell count, neutrophils, lymphocytes, platelets, creatinine, urea, liver enzymes, bilirubin, electrolytes, glucose, osmolality, lipids, coagulation tests |
| Nutrition and gastrointestinal management | Enteral formula type, feeding dose, enteral infusion rate, fasting duration categories, prokinetic therapy, prophylactic laxative use, parenteral nutrition |
| Medication and organ support | Antibiotic therapy, vasoactive drugs, sedative agents, analgesic agents, oxygen therapy, mechanical ventilation duration, fluid balance |
| Stroke-related characteristics | Stroke location/position, treatment/intervention type, ICU length of stay |

EN, enteral nutrition; ICU, intensive care unit.

**2.2 Missing-data assessment and handling**

Missingness was quantified separately in the raw data-collection workbook and in the final analytic dataset before model development. Patient identifiers and direct patient-level examples were not included in the supplementary outputs. Missing values were handled within the cross-validation training folds: median imputation for continuous predictors and most-frequent imputation for categorical predictors. Outcome values were not imputed, and variables considered potential post-outcome or leakage variables were excluded from model development.

**2.3 Internal validation and hyperparameter reporting**

The primary internal-validation results were based on 5-fold cross-validated out-of-fold predictions. Within each fold, imputation and any required preprocessing were estimated in the training fold and applied to the validation fold. Prespecified conservative hyperparameter values were applied consistently across folds to reduce overfitting in the limited sample; effective final hyperparameters, implemented candidate search spaces, and internal-CV penalized ROC-AUC values are reported below to improve reproducibility.

**2.4 Collinearity, neuroseverity redundancy, and SHAP-stability analyses**

Collinearity was examined using Spearman correlation and variance inflation factors (VIFs). Sensitivity analyses replaced neurological/severity scores with a first principal component or reduced the neurological-score cluster. SHAP-rank stability was examined using bootstrap resampling of the random forest model.

**2.5 Parsimonious and Liao-compatible models**

To address clinical usability, the full model was compared with a study-specific 12-predictor model and a Liao-compatible overlapping-predictor model. Exact replication of the Liao et al. 12-predictor model was not possible because several variables were not collected in this cohort.

**3. Supplementary tables**

**Supplementary Table S2A. Raw data-collection workbook sheet summary.**

| **Source sheet** | **Denominator n** | **Columns detected** | **Notes** |
| --- | --- | --- | --- |
| 二病区1-9月 | 382 | 86 | Header rows=1; empty columns excluded from table |
| Sheet2 | 176 | 68 | Header rows=2; empty columns excluded from table |
| Sheet3 | 0 | 0 | Empty sheet; not analyzed |

Denominators were calculated after excluding header rows and empty columns. The primary raw data sheet contained 382 potentially eligible EN records; after final eligibility confirmation, de-duplication, and data-quality review, 374 records were retained for the analytic cohort reported in the manuscript.

**Supplementary Table S2B. Missingness and handling of model-development predictors in the final analytic dataset (n = 374).**

| **Predictor** | **Domain** | **Data type** | **Non-missing n** | **Missing n** | **Missing %** | **Handling strategy** |
| --- | --- | --- | --- | --- | --- | --- |
| Gender | Demographics/lifestyle | Categorical/ordinal numeric | 374 | 0 | 0.00 | Most-frequent imputation within CV training folds. |
| Age | Demographics/lifestyle | Continuous numeric | 374 | 0 | 0.00 | Median imputation within CV training folds. |
| NRS2002 | Neurological/critical-illness severity | Continuous numeric | 374 | 0 | 0.00 | Median imputation within CV training folds. |
| BMI | Demographics/lifestyle | Continuous numeric | 374 | 0 | 0.00 | Median imputation within CV training folds. |
| Position | Other clinical variable | Categorical/ordinal numeric | 374 | 0 | 0.00 | Most-frequent imputation within CV training folds. |
| Treatment | Other clinical variable | Categorical/ordinal numeric | 373 | 1 | 0.27 | Most-frequent imputation within CV training folds. |
| APACHE2 | Neurological/critical-illness severity | Continuous numeric | 374 | 0 | 0.00 | Median imputation within CV training folds. |
| GCS | Neurological/critical-illness severity | Continuous numeric | 374 | 0 | 0.00 | Median imputation within CV training folds. |
| MRS | Neurological/critical-illness severity | Continuous numeric | 374 | 0 | 0.00 | Median imputation within CV training folds. |
| NIHSS | Neurological/critical-illness severity | Continuous numeric | 374 | 0 | 0.00 | Median imputation within CV training folds. |
| CV | Other clinical variable | Categorical/ordinal numeric | 374 | 0 | 0.00 | Most-frequent imputation within CV training folds. |
| Fluid_Balance | Other clinical variable | Continuous numeric | 374 | 0 | 0.00 | Median imputation within CV training folds. |
| Hypertension | Comorbidity | Categorical/ordinal numeric | 374 | 0 | 0.00 | Most-frequent imputation within CV training folds. |
| Smoking_Status | Demographics/lifestyle | Categorical/ordinal numeric | 374 | 0 | 0.00 | Most-frequent imputation within CV training folds. |
| Alcohol_Drinker | Demographics/lifestyle | Categorical/ordinal numeric | 374 | 0 | 0.00 | Most-frequent imputation within CV training folds. |
| Fasting_Within_24h | Enteral nutrition / feeding process | Categorical/ordinal numeric | 374 | 0 | 0.00 | Most-frequent imputation within CV training folds. |
| Fasting_24h_to_48h | Enteral nutrition / feeding process | Categorical/ordinal numeric | 374 | 0 | 0.00 | Most-frequent imputation within CV training folds. |
| Enteral_Formula_Type | Enteral nutrition / feeding process | Categorical/ordinal numeric | 374 | 0 | 0.00 | Most-frequent imputation within CV training folds. |
| Enteral_Infusion_Rate | Enteral nutrition / feeding process | Continuous numeric | 374 | 0 | 0.00 | Median imputation within CV training folds. |
| Antibiotic_Therapy | Medication/treatment exposure | Categorical/ordinal numeric | 374 | 0 | 0.00 | Most-frequent imputation within CV training folds. |
| Prokinetic_Therapy | Medication/treatment exposure | Categorical/ordinal numeric | 374 | 0 | 0.00 | Most-frequent imputation within CV training folds. |
| Prophylactic_Laxative_Use | Medication/treatment exposure | Categorical/ordinal numeric | 374 | 0 | 0.00 | Most-frequent imputation within CV training folds. |
| Daily_Potassium_Dose | Medication/treatment exposure | Continuous numeric | 374 | 0 | 0.00 | Median imputation within CV training folds. |
| Sedative_Agent | Demographics/lifestyle | Categorical/ordinal numeric | 374 | 0 | 0.00 | Most-frequent imputation within CV training folds. |
| Analgesic_Agent | Demographics/lifestyle | Categorical/ordinal numeric | 374 | 0 | 0.00 | Most-frequent imputation within CV training folds. |
| T | Other clinical variable | Continuous numeric | 374 | 0 | 0.00 | Median imputation within CV training folds. |
| P | Other clinical variable | Continuous numeric | 374 | 0 | 0.00 | Median imputation within CV training folds. |
| R | Other clinical variable | Continuous numeric | 374 | 0 | 0.00 | Median imputation within CV training folds. |
| MAP | Other clinical variable | Continuous numeric | 374 | 0 | 0.00 | Median imputation within CV training folds. |
| RASS | Neurological/critical-illness severity | Continuous numeric | 372 | 2 | 0.53 | Median imputation within CV training folds. |
| pH | Arterial blood gas/electrolyte/metabolic | Continuous numeric | 374 | 0 | 0.00 | Median imputation within CV training folds. |
| tHb | Arterial blood gas/electrolyte/metabolic | Continuous numeric | 374 | 0 | 0.00 | Median imputation within CV training folds. |
| K | Other clinical variable | Continuous numeric | 374 | 0 | 0.00 | Median imputation within CV training folds. |
| Na | Arterial blood gas/electrolyte/metabolic | Continuous numeric | 374 | 0 | 0.00 | Median imputation within CV training folds. |
| Cl | Arterial blood gas/electrolyte/metabolic | Continuous numeric | 374 | 0 | 0.00 | Median imputation within CV training folds. |
| Ca | Arterial blood gas/electrolyte/metabolic | Continuous numeric | 374 | 0 | 0.00 | Median imputation within CV training folds. |
| Glu | Arterial blood gas/electrolyte/metabolic | Continuous numeric | 374 | 0 | 0.00 | Median imputation within CV training folds. |
| Lac | Arterial blood gas/electrolyte/metabolic | Continuous numeric | 374 | 0 | 0.00 | Median imputation within CV training folds. |
| HCO3 | Arterial blood gas/electrolyte/metabolic | Continuous numeric | 374 | 0 | 0.00 | Median imputation within CV training folds. |
| BB | Arterial blood gas/electrolyte/metabolic | Continuous numeric | 374 | 0 | 0.00 | Median imputation within CV training folds. |
| BE | Arterial blood gas/electrolyte/metabolic | Continuous numeric | 374 | 0 | 0.00 | Median imputation within CV training folds. |
| AG | Arterial blood gas/electrolyte/metabolic | Continuous numeric | 374 | 0 | 0.00 | Median imputation within CV training folds. |
| Osm | Arterial blood gas/electrolyte/metabolic | Continuous numeric | 374 | 0 | 0.00 | Median imputation within CV training folds. |
| SaO2 | Respiratory support/oxygenation | Continuous numeric | 374 | 0 | 0.00 | Median imputation within CV training folds. |
| PF_Ratio | Respiratory support/oxygenation | Continuous numeric | 374 | 0 | 0.00 | Median imputation within CV training folds. |
| WBC | Hematology/inflammation | Continuous numeric | 374 | 0 | 0.00 | Median imputation within CV training folds. |
| HGB | Hematology/inflammation | Continuous numeric | 374 | 0 | 0.00 | Median imputation within CV training folds. |
| PLT | Hematology/inflammation | Continuous numeric | 374 | 0 | 0.00 | Median imputation within CV training folds. |
| NEUT | Hematology/inflammation | Continuous numeric | 353 | 21 | 5.61 | Median imputation within CV training folds. |
| LYM | Hematology/inflammation | Continuous numeric | 374 | 0 | 0.00 | Median imputation within CV training folds. |
| TC | Biochemistry/liver/renal/lipid/coagulation | Continuous numeric | 299 | 75 | 20.05 | Median imputation within CV training folds. |
| TG | Biochemistry/liver/renal/lipid/coagulation | Continuous numeric | 299 | 75 | 20.05 | Median imputation within CV training folds. |
| HDL | Biochemistry/liver/renal/lipid/coagulation | Continuous numeric | 299 | 75 | 20.05 | Median imputation within CV training folds. |
| LDL | Biochemistry/liver/renal/lipid/coagulation | Continuous numeric | 299 | 75 | 20.05 | Median imputation within CV training folds. |
| Urea | Biochemistry/liver/renal/lipid/coagulation | Continuous numeric | 371 | 3 | 0.80 | Median imputation within CV training folds. |
| CRE | Biochemistry/liver/renal/lipid/coagulation | Continuous numeric | 371 | 3 | 0.80 | Median imputation within CV training folds. |
| CRP | Hematology/inflammation | Continuous numeric | 373 | 1 | 0.27 | Median imputation within CV training folds. |
| ALT | Biochemistry/liver/renal/lipid/coagulation | Continuous numeric | 371 | 3 | 0.80 | Median imputation within CV training folds. |
| AST | Biochemistry/liver/renal/lipid/coagulation | Continuous numeric | 372 | 2 | 0.53 | Median imputation within CV training folds. |
| TBIL | Biochemistry/liver/renal/lipid/coagulation | Continuous numeric | 370 | 4 | 1.07 | Median imputation within CV training folds. |
| DBIL | Biochemistry/liver/renal/lipid/coagulation | Continuous numeric | 370 | 4 | 1.07 | Median imputation within CV training folds. |
| ALB | Biochemistry/liver/renal/lipid/coagulation | Continuous numeric | 374 | 0 | 0.00 | Median imputation within CV training folds. |
| PT | Biochemistry/liver/renal/lipid/coagulation | Continuous numeric | 362 | 12 | 3.21 | Median imputation within CV training folds. |
| Oxygen_Therapy | Respiratory support/oxygenation | Categorical/ordinal numeric | 374 | 0 | 0.00 | Most-frequent imputation within CV training folds. |
| FiO2 | Respiratory support/oxygenation | Continuous numeric | 374 | 0 | 0.00 | Median imputation within CV training folds. |

The table is anonymized and reports variable-level missingness only. Numeric zero was not treated as missing.

**Supplementary Table S2C. Raw data-collection variables/items with non-zero missingness.**

| **Source sheet** | **Variable/item** | **Domain** | **Missing n** | **Missing %** | **Missingness category** | **Handling strategy** |
| --- | --- | --- | --- | --- | --- | --- |
| 二病区1-9月 | HbA1c | Other clinical variable | 273 | 71.47 | High (>20%) | Excluded/not retained in final prediction model. |
| 二病区1-9月 | VTE | Other clinical variable | 203 | 53.14 | High (>20%) | Excluded/not retained in final prediction model. |
| 二病区1-9月 | Ventilator_Mode | Respiratory support/oxygenation | 202 | 52.88 | High (>20%) | Excluded/not retained in final prediction model. |
| 二病区1-9月 | PEEP | Respiratory support/oxygenation | 202 | 52.88 | High (>20%) | Excluded/not retained in final prediction model. |
| 二病区1-9月 | PAB | Biochemistry/liver/renal/lipid/coagulation | 178 | 46.60 | High (>20%) | Excluded/not retained in final prediction model. |
| 二病区1-9月 | TC | Biochemistry/liver/renal/lipid/coagulation | 77 | 20.16 | High (>20%) | Median imputation within CV training folds. |
| 二病区1-9月 | TG | Biochemistry/liver/renal/lipid/coagulation | 77 | 20.16 | High (>20%) | Median imputation within CV training folds. |
| 二病区1-9月 | HDL | Biochemistry/liver/renal/lipid/coagulation | 77 | 20.16 | High (>20%) | Median imputation within CV training folds. |
| 二病区1-9月 | LDL | Biochemistry/liver/renal/lipid/coagulation | 77 | 20.16 | High (>20%) | Median imputation within CV training folds. |
| 二病区1-9月 | NEUT | Hematology/inflammation | 21 | 5.50 | Moderate (>5-20%) | Median imputation within CV training folds. |
| 二病区1-9月 | PT | Biochemistry/liver/renal/lipid/coagulation | 12 | 3.14 | Low (<=5%) | Median imputation within CV training folds. |
| 二病区1-9月 | TBIL | Biochemistry/liver/renal/lipid/coagulation | 4 | 1.05 | Low (<=5%) | Median imputation within CV training folds. |
| 二病区1-9月 | DBIL | Biochemistry/liver/renal/lipid/coagulation | 4 | 1.05 | Low (<=5%) | Median imputation within CV training folds. |
| 二病区1-9月 | Urea | Biochemistry/liver/renal/lipid/coagulation | 3 | 0.79 | Low (<=5%) | Median imputation within CV training folds. |
| 二病区1-9月 | CRE | Biochemistry/liver/renal/lipid/coagulation | 3 | 0.79 | Low (<=5%) | Median imputation within CV training folds. |
| 二病区1-9月 | ALT | Biochemistry/liver/renal/lipid/coagulation | 3 | 0.79 | Low (<=5%) | Median imputation within CV training folds. |
| 二病区1-9月 | RASS | Neurological/critical-illness severity | 2 | 0.52 | Low (<=5%) | Median imputation within CV training folds. |
| 二病区1-9月 | AST | Biochemistry/liver/renal/lipid/coagulation | 2 | 0.52 | Low (<=5%) | Median imputation within CV training folds. |
| 二病区1-9月 | Treatment | Other clinical variable | 1 | 0.26 | Low (<=5%) | Most-frequent imputation within CV training folds. |
| 二病区1-9月 | CRP | Hematology/inflammation | 1 | 0.26 | Low (<=5%) | Median imputation within CV training folds. |
| Sheet2 | 肠内营养48小时 - 第一次 | Enteral nutrition / feeding process | 176 | 100.00 | Completely missing (100%) | Not collected/not available; not used for model development. |
| Sheet2 | 肠内营养48小时 - 第二次 | Enteral nutrition / feeding process | 176 | 100.00 | Completely missing (100%) | Not collected/not available; not used for model development. |
| Sheet2 | 肠内营养48小时 - 第三次 | Enteral nutrition / feeding process | 176 | 100.00 | Completely missing (100%) | Not collected/not available; not used for model development. |
| Sheet2 | 肠内营养48小时 - 第四次 | Enteral nutrition / feeding process | 176 | 100.00 | Completely missing (100%) | Not collected/not available; not used for model development. |
| Sheet2 | 肠内营养48小时 - 第五次 | Enteral nutrition / feeding process | 176 | 100.00 | Completely missing (100%) | Not collected/not available; not used for model development. |
| Sheet2 | 肠内营养48小时 - 第六次 | Enteral nutrition / feeding process | 176 | 100.00 | Completely missing (100%) | Not collected/not available; not used for model development. |
| Sheet2 | 肠内营养48小时 - 均值 | Enteral nutrition / feeding process | 176 | 100.00 | Completely missing (100%) | Not collected/not available; not used for model development. |
| Sheet2 | 肠内营养48小时 - 变异系数 | Enteral nutrition / feeding process | 176 | 100.00 | Completely missing (100%) | Not collected/not available; not used for model development. |
| Sheet2 | 肠内营养48小时 - 高血糖次数 | Enteral nutrition / feeding process | 176 | 100.00 | Completely missing (100%) | Not collected/not available; not used for model development. |
| Sheet2 | 肠内营养48小时 - 胰岛素剂量 | Enteral nutrition / feeding process | 176 | 100.00 | Completely missing (100%) | Not collected/not available; not used for model development. |
| Sheet2 | 肠内营养48小时 - 肠内营养时间 | Enteral nutrition / feeding process | 176 | 100.00 | Completely missing (100%) | Not collected/not available; not used for model development. |
| Sheet2 | 肠内营养48小时 - 营养总量 | Enteral nutrition / feeding process | 176 | 100.00 | Completely missing (100%) | Not collected/not available; not used for model development. |
| Sheet2 | 肠内营养48小时 - 碳水化合物摄入量 | Enteral nutrition / feeding process | 176 | 100.00 | Completely missing (100%) | Not collected/not available; not used for model development. |
| Sheet2 | 肠内营养48小时 - 营养种类 | Enteral nutrition / feeding process | 176 | 100.00 | Completely missing (100%) | Not collected/not available; not used for model development. |
| Sheet2 | 肠内营养48小时 - 输注速度 | Enteral nutrition / feeding process | 176 | 100.00 | Completely missing (100%) | Not collected/not available; not used for model development. |
| Sheet2 | 肠内营养48小时 - 输注途径 | Enteral nutrition / feeding process | 176 | 100.00 | Completely missing (100%) | Not collected/not available; not used for model development. |
| Sheet2 | 肠内营养48小时 - 高密度脂蛋白 | Enteral nutrition / feeding process | 176 | 100.00 | Completely missing (100%) | Not collected/not available; not used for model development. |
| Sheet2 | 肠内营养48小时 - 低密度脂蛋白 | Enteral nutrition / feeding process | 176 | 100.00 | Completely missing (100%) | Not collected/not available; not used for model development. |
| Sheet2 | 肠内营养48小时 - 胆固醇 | Enteral nutrition / feeding process | 176 | 100.00 | Completely missing (100%) | Not collected/not available; not used for model development. |
| Sheet2 | 肠内营养48小时 - 甘油三酯 | Enteral nutrition / feeding process | 176 | 100.00 | Completely missing (100%) | Not collected/not available; not used for model development. |
| Sheet2 | 肠内营养48小时 - PCT | Enteral nutrition / feeding process | 176 | 100.00 | Completely missing (100%) | Not collected/not available; not used for model development. |
| Sheet2 | 肠内营养72小时 - 第一次 | Enteral nutrition / feeding process | 176 | 100.00 | Completely missing (100%) | Not collected/not available; not used for model development. |
| Sheet2 | 肠内营养72小时 - 第二次 | Enteral nutrition / feeding process | 176 | 100.00 | Completely missing (100%) | Not collected/not available; not used for model development. |
| Sheet2 | 肠内营养72小时 - 第三次 | Enteral nutrition / feeding process | 176 | 100.00 | Completely missing (100%) | Not collected/not available; not used for model development. |
| Sheet2 | 肠内营养72小时 - 第四次 | Enteral nutrition / feeding process | 176 | 100.00 | Completely missing (100%) | Not collected/not available; not used for model development. |
| Sheet2 | 肠内营养72小时 - 第五次 | Enteral nutrition / feeding process | 176 | 100.00 | Completely missing (100%) | Not collected/not available; not used for model development. |
| Sheet2 | 肠内营养72小时 - 第六次 | Enteral nutrition / feeding process | 176 | 100.00 | Completely missing (100%) | Not collected/not available; not used for model development. |
| Sheet2 | 肠内营养72小时 - 均值 | Enteral nutrition / feeding process | 176 | 100.00 | Completely missing (100%) | Not collected/not available; not used for model development. |
| Sheet2 | 肠内营养72小时 - 变异系数 | Enteral nutrition / feeding process | 176 | 100.00 | Completely missing (100%) | Not collected/not available; not used for model development. |
| Sheet2 | 肠内营养72小时 - 高血糖次数 | Enteral nutrition / feeding process | 176 | 100.00 | Completely missing (100%) | Not collected/not available; not used for model development. |
| Sheet2 | 肠内营养72小时 - 胰岛素剂量 | Enteral nutrition / feeding process | 176 | 100.00 | Completely missing (100%) | Not collected/not available; not used for model development. |
| Sheet2 | 肠内营养72小时 - 肠内营养时间 | Enteral nutrition / feeding process | 176 | 100.00 | Completely missing (100%) | Not collected/not available; not used for model development. |
| Sheet2 | 肠内营养72小时 - 营养总量 | Enteral nutrition / feeding process | 176 | 100.00 | Completely missing (100%) | Not collected/not available; not used for model development. |
| Sheet2 | 肠内营养72小时 - 碳水化合物摄入量 | Enteral nutrition / feeding process | 176 | 100.00 | Completely missing (100%) | Not collected/not available; not used for model development. |
| Sheet2 | 肠内营养72小时 - 营养种类 | Enteral nutrition / feeding process | 176 | 100.00 | Completely missing (100%) | Not collected/not available; not used for model development. |
| Sheet2 | 肠内营养72小时 - 输注速度 | Enteral nutrition / feeding process | 176 | 100.00 | Completely missing (100%) | Not collected/not available; not used for model development. |
| Sheet2 | 肠内营养72小时 - 输注途径 | Enteral nutrition / feeding process | 176 | 100.00 | Completely missing (100%) | Not collected/not available; not used for model development. |
| Sheet2 | 肠内营养72小时 - 高密度脂蛋白 | Enteral nutrition / feeding process | 176 | 100.00 | Completely missing (100%) | Not collected/not available; not used for model development. |
| Sheet2 | 肠内营养72小时 - 低密度脂蛋白 | Enteral nutrition / feeding process | 176 | 100.00 | Completely missing (100%) | Not collected/not available; not used for model development. |
| Sheet2 | 肠内营养72小时 - 胆固醇 | Enteral nutrition / feeding process | 176 | 100.00 | Completely missing (100%) | Not collected/not available; not used for model development. |
| Sheet2 | 肠内营养72小时 - 甘油三酯 | Enteral nutrition / feeding process | 176 | 100.00 | Completely missing (100%) | Not collected/not available; not used for model development. |
| Sheet2 | 肠内营养72小时 - 尿酸 | Enteral nutrition / feeding process | 174 | 98.86 | High (>20%) | Excluded/not retained in final prediction model. |
| Sheet2 | 肠内营养72小时 - PCT | Enteral nutrition / feeding process | 172 | 97.73 | High (>20%) | Excluded/not retained in final prediction model. |
| Sheet2 | 肠内营养48小时 - 尿酸 | Enteral nutrition / feeding process | 171 | 97.16 | High (>20%) | Excluded/not retained in final prediction model. |
| Sheet2 | 肠内营养72小时 - 谷丙转氨酶 | Enteral nutrition / feeding process | 73 | 41.48 | High (>20%) | Excluded/not retained in final prediction model. |
| Sheet2 | 肠内营养72小时 - 总蛋白 | Enteral nutrition / feeding process | 72 | 40.91 | High (>20%) | Excluded/not retained in final prediction model. |
| Sheet2 | 肠内营养72小时 - 白蛋白 | Enteral nutrition / feeding process | 71 | 40.34 | High (>20%) | Excluded/not retained in final prediction model. |
| Sheet2 | 肠内营养72小时 - 谷草转氨酶 | Enteral nutrition / feeding process | 70 | 39.77 | High (>20%) | Excluded/not retained in final prediction model. |
| Sheet2 | 肠内营养72小时 - 尿素氮 | Enteral nutrition / feeding process | 70 | 39.77 | High (>20%) | Excluded/not retained in final prediction model. |
| Sheet2 | 肠内营养72小时 - 肌酐 | Enteral nutrition / feeding process | 68 | 38.64 | High (>20%) | Excluded/not retained in final prediction model. |
| Sheet2 | 肠内营养72小时 - 白细胞 | Enteral nutrition / feeding process | 54 | 30.68 | High (>20%) | Excluded/not retained in final prediction model. |
| Sheet2 | 肠内营养72小时 - 血红蛋白 | Enteral nutrition / feeding process | 53 | 30.11 | High (>20%) | Excluded/not retained in final prediction model. |
| Sheet2 | 肠内营养72小时 - 中性粒细胞 | Enteral nutrition / feeding process | 53 | 30.11 | High (>20%) | Excluded/not retained in final prediction model. |
| Sheet2 | 肠内营养72小时 - 淋巴细胞 | Enteral nutrition / feeding process | 53 | 30.11 | High (>20%) | Excluded/not retained in final prediction model. |
| Sheet2 | 肠内营养72小时 - CRP | Enteral nutrition / feeding process | 52 | 29.55 | High (>20%) | Median imputation within CV training folds. |
| Sheet2 | 肠内营养48小时 - 尿素氮 | Enteral nutrition / feeding process | 48 | 27.27 | High (>20%) | Excluded/not retained in final prediction model. |
| Sheet2 | 肠内营养48小时 - 肌酐 | Enteral nutrition / feeding process | 47 | 26.70 | High (>20%) | Excluded/not retained in final prediction model. |
| Sheet2 | 肠内营养48小时 - 谷草转氨酶 | Enteral nutrition / feeding process | 39 | 22.16 | High (>20%) | Excluded/not retained in final prediction model. |
| Sheet2 | 肠内营养48小时 - 谷丙转氨酶 | Enteral nutrition / feeding process | 39 | 22.16 | High (>20%) | Excluded/not retained in final prediction model. |
| Sheet2 | 肠内营养48小时 - 总蛋白 | Enteral nutrition / feeding process | 39 | 22.16 | High (>20%) | Excluded/not retained in final prediction model. |
| Sheet2 | 肠内营养48小时 - 白蛋白 | Enteral nutrition / feeding process | 38 | 21.59 | High (>20%) | Excluded/not retained in final prediction model. |
| Sheet2 | 肠内营养48小时 - CRP | Enteral nutrition / feeding process | 31 | 17.61 | Moderate (>5-20%) | Median imputation within CV training folds. |
| Sheet2 | 肠内营养48小时 - 白细胞 | Enteral nutrition / feeding process | 29 | 16.48 | Moderate (>5-20%) | Not included in final analytic dataset; excluded before model development. |
| Sheet2 | 肠内营养48小时 - 血红蛋白 | Enteral nutrition / feeding process | 27 | 15.34 | Moderate (>5-20%) | Not included in final analytic dataset; excluded before model development. |
| Sheet2 | 肠内营养48小时 - 中性粒细胞 | Enteral nutrition / feeding process | 27 | 15.34 | Moderate (>5-20%) | Not included in final analytic dataset; excluded before model development. |
| Sheet2 | 肠内营养48小时 - 淋巴细胞 | Enteral nutrition / feeding process | 27 | 15.34 | Moderate (>5-20%) | Not included in final analytic dataset; excluded before model development. |

This table summarizes non-zero missingness in the raw data-collection workbook. Variables not collected or with high missingness were not used for model development unless already included in the finalized analytic predictor set and handled within cross-validation folds.

**Supplementary Table S3A. Hyperparameter specification and implemented search spaces for all six candidate algorithms.**

| **Model** | **Effective final hyperparameters** | **Internal-CV penalized ROC-AUC** | **Implemented candidate search space / selection strategy** | **Reproducibility notes** |
| --- | --- | --- | --- | --- |
| Regularized logistic regression | selector=L1 logistic SelectFromModel(C=0.2, threshold=median); classifier: LogisticRegression(penalty=l2, C=1.0, solver=liblinear, class_weight=balanced, max_iter=5000, random_state=fold seed) | mean=0.955; range=0.940-0.972 | Optuna: selector none/L1; selector_C 0.01-10 log; penalty l1/l2; C 0.005-50 log. Small grid: penalty l1/l2 × C 0.01/0.05/0.2/1/5/20 with optional L1 selector C 0.05/0.2/1/5. | Applied consistently across the five cross-validation folds with fold-specific random seeds; preprocessing and class-imbalance handling were estimated within training folds only. |
| Support vector machine | selector=L1 logistic SelectFromModel(C=0.2, threshold=median); classifier: SVC(kernel=rbf, C=1.0, gamma=0.01, class_weight=balanced, probability=True, cache_size=1000, random_state=fold seed) | mean=0.963; range=0.952-0.974 | Optuna: selector none/L1; selector_C 0.01-10 log; C 0.01-50 log; gamma 1e-4-1.0 log. Small grid: C 0.05/0.2/1/5/20 × gamma 0.001/0.01/0.05/0.2 with optional L1 selector. | Applied consistently across the five cross-validation folds with fold-specific random seeds; preprocessing and class-imbalance handling were estimated within training folds only. |
| Random forest | RandomForestClassifier(n_estimators=400, max_depth=3, min_samples_leaf=5, min_samples_split=10, max_features=sqrt, class_weight=balanced_subsample, bootstrap=True, n_jobs=-1, random_state=fold seed) | mean=0.972; range=0.967-0.979 | Optuna: n_estimators 200-800 step100; max_depth 2/3/4/5/6/None; min_samples_leaf 2-20; min_samples_split 4-30; max_features sqrt/log2/0.3/0.5/0.8. Small grid: n_estimators=400; max_depth 2/3/4/None; leaf 2/5/10/15; max_features sqrt/0.5. | Applied consistently across the five cross-validation folds with fold-specific random seeds; preprocessing and class-imbalance handling were estimated within training folds only. |
| Extra trees | ExtraTreesClassifier(n_estimators=500, max_depth=3, min_samples_leaf=5, min_samples_split=10, max_features=sqrt, class_weight=balanced, n_jobs=-1, random_state=fold seed) | mean=0.947; range=0.939-0.962 | Optuna: n_estimators 200-800 step100; max_depth 2/3/4/5/6/None; min_samples_leaf 2-20; min_samples_split 4-30; max_features sqrt/log2/0.3/0.5/0.8. Small grid: n_estimators=500; max_depth 2/3/4/None; leaf 2/5/10/15; max_features sqrt/0.5. | Applied consistently across the five cross-validation folds with fold-specific random seeds; preprocessing and class-imbalance handling were estimated within training folds only. |
| XGBoost | XGBClassifier(objective=binary:logistic, eval_metric=logloss, tree_method=hist, n_estimators=300, max_depth=2, learning_rate=0.05, subsample=0.8, colsample_bytree=0.8, min_child_weight=2.0, reg_alpha=0.01, reg_lambda=5.0, scale_pos_weight=neg/pos within training fold, random_state=fold seed, n_jobs=-1) | mean=0.967; range=0.961-0.973 | Optuna: n_estimators 100-700 step50; max_depth 1-4; learning_rate 0.005-0.15 log; subsample 0.55-1.0; colsample_bytree 0.45-1.0; min_child_weight 1-20 log; reg_alpha 1e-8-5 log; reg_lambda 0.1-20 log. Small grid: n 150/300/500 × depth 1/2/3 × lr 0.02/0.05 × min_child_weight 2/8, with subsample/colsample=0.8, alpha=0.01, lambda=5.0. | Applied consistently across the five cross-validation folds with fold-specific random seeds; preprocessing and class-imbalance handling were estimated within training folds only. |
| Gradient boosting | GradientBoostingClassifier(n_estimators=200, learning_rate=0.05, max_depth=2, subsample=0.8, min_samples_leaf=5, random_state=fold seed) | mean=0.969; range=0.961-0.977 | Optuna: n_estimators 50-500 step50; learning_rate 0.005-0.15 log; max_depth 1-3; subsample 0.55-1.0; min_samples_leaf 2-30. Small grid: n 100/200/400 × lr 0.02/0.05 × depth 1/2 × leaf 5/15, subsample=0.8. | Applied consistently across the five cross-validation folds with fold-specific random seeds; preprocessing and class-imbalance handling were estimated within training folds only. |

The final hyperparameter values were applied consistently across the five cross-validation folds. Implemented candidate search spaces and internal-CV penalized ROC-AUC values are shown to improve reproducibility and to clarify the model-building strategy for all six candidate algorithms.

**Supplementary Table S3B. Cross-validated internal performance and confusion-matrix counts for the six candidate algorithms.**

| **Model** | **AUC (95% CI)** | **AUPRC (95% CI)** | **Brier (95% CI)** | **Accuracy (95% CI)** | **Sensitivity (95% CI)** | **Specificity (95% CI)** | **PPV (95% CI)** | **NPV (95% CI)** | **F1 (95% CI)** | **Threshold** | **TP** | **FP** | **TN** | **FN** |
| --- | --- | --- | --- | --- | --- | --- | --- | --- | --- | --- | --- | --- | --- | --- |
| Random forest | 0.969 (0.951-0.982) | 0.921 (0.877-0.957) | 0.123 (0.114-0.133) | 0.904 (0.872-0.933) | 0.943 (0.892-0.982) | 0.888 (0.851-0.921) | 0.767 (0.692-0.836) | 0.976 (0.952-0.992) | 0.846 (0.794-0.892) | 0.451 | 99 | 30 | 239 | 6 |
| XGBoost | 0.968 (0.949-0.983) | 0.919 (0.870-0.959) | 0.062 (0.046-0.080) | 0.906 (0.874-0.933) | 0.933 (0.880-0.979) | 0.896 (0.861-0.929) | 0.778 (0.705-0.851) | 0.972 (0.951-0.992) | 0.848 (0.795-0.897) | 0.289 | 98 | 28 | 241 | 7 |
| Gradient boosting | 0.966 (0.947-0.982) | 0.919 (0.873-0.960) | 0.066 (0.049-0.087) | 0.885 (0.852-0.917) | 0.924 (0.871-0.970) | 0.870 (0.835-0.909) | 0.735 (0.662-0.810) | 0.967 (0.944-0.988) | 0.819 (0.758-0.872) | 0.138 | 97 | 35 | 234 | 8 |
| Support vector machine | 0.965 (0.947-0.981) | 0.924 (0.883-0.960) | 0.064 (0.049-0.082) | 0.901 (0.869-0.932) | 0.886 (0.821-0.946) | 0.907 (0.872-0.939) | 0.788 (0.716-0.860) | 0.953 (0.925-0.977) | 0.834 (0.779-0.888) | 0.333 | 93 | 25 | 244 | 12 |
| Regularized logistic regression | 0.960 (0.939-0.976) | 0.912 (0.862-0.950) | 0.074 (0.054-0.098) | 0.869 (0.835-0.902) | 0.924 (0.876-0.963) | 0.848 (0.805-0.890) | 0.703 (0.630-0.781) | 0.966 (0.941-0.984) | 0.798 (0.741-0.852) | 0.205 | 97 | 41 | 228 | 8 |
| Extra trees | 0.950 (0.926-0.969) | 0.891 (0.840-0.935) | 0.158 (0.150-0.166) | 0.840 (0.802-0.877) | 0.952 (0.903-0.990) | 0.796 (0.746-0.846) | 0.645 (0.575-0.728) | 0.977 (0.952-0.995) | 0.769 (0.710-0.829) | 0.449 | 100 | 55 | 214 | 5 |

Performance estimates are based on 5-fold cross-validated out-of-fold predictions. TP, true positives; FP, false positives; TN, true negatives; FN, false negatives; PPV, positive predictive value; NPV, negative predictive value.

**Supplementary Table S4. Variables retained after feature screening and ranked by aggregated native-model importance.**

| **Rank** | **Predictor** |
| --- | --- |
| 1 | Enteral infusion rate |
| 2 | Albumin |
| 3 | mRS |
| 4 | NRS-2002 |
| 5 | CRP |
| 6 | APACHE II |
| 7 | NIHSS |
| 8 | Hemoglobin |
| 9 | Daily potassium dose |
| 10 | GCS |
| 11 | CV |
| 12 | FiO2 |
| 13 | Lactate |
| 14 | PaO2/FiO2 ratio |
| 15 | Lymphocytes |
| 16 | Total hemoglobin |
| 17 | Glucose |
| 18 | Calcium |
| 19 | SaO2 |
| 20 | White blood cell count |

Predictor names were standardized for readability in the manuscript.

**Supplementary Table S5. Hemoglobin (HGB) and total hemoglobin (tHb) duplicate/near-duplicate assessment.**

| **Both non-missing n** | **HGB missing n** | **tHb missing n** | **Pearson r: HGB vs tHb×10** | **Spearman rho: HGB vs tHb×10** | **Mean difference HGB - tHb×10** | **Median difference HGB - tHb×10** | **Median absolute difference** | **Decision** |
| --- | --- | --- | --- | --- | --- | --- | --- | --- |
| 374 | 0 | 0 | 0.842 | 0.838 | -3.460 | -1.900 | 4.200 | distinct enough for retention with collinearity diagnostics |

HGB represents routine hemoglobin; tHb represents total hemoglobin. Because HGB and tHb×10 did not meet the prespecified near-duplicate threshold, both variables were retained and assessed in collinearity diagnostics.

**Supplementary Table S6. Variance inflation factor diagnostics for selected final-model predictors.**

| **Feature** | **VIF** | **Interpretation** |
| --- | --- | --- |
| HGB | 3.84 | low/moderate collinearity |
| tHb | 3.68 | low/moderate collinearity |
| PF_Ratio | 1.91 | low/moderate collinearity |
| SaO2 | 1.89 | low/moderate collinearity |
| CRP | 1.42 | low/moderate collinearity |
| ALB | 1.42 | low/moderate collinearity |
| NIHSS | 1.37 | low/moderate collinearity |
| GCS | 1.33 | low/moderate collinearity |
| MRS | 1.27 | low/moderate collinearity |
| Lac | 1.27 | low/moderate collinearity |
| NRS2002 | 1.25 | low/moderate collinearity |
| Glu | 1.24 | low/moderate collinearity |
| CV | 1.20 | low/moderate collinearity |
| WBC | 1.18 | low/moderate collinearity |
| APACHE2 | 1.18 | low/moderate collinearity |
| Enteral_Infusion_Rate | 1.17 | low/moderate collinearity |
| FiO2 | 1.11 | low/moderate collinearity |
| Daily_Potassium_Dose | 1.11 | low/moderate collinearity |
| Ca | 1.11 | low/moderate collinearity |
| LYM | 1.11 | low/moderate collinearity |

VIF < 5 was interpreted as low/moderate collinearity; VIF 5-10 as potential collinearity; and VIF > 10 as serious collinearity.

**Supplementary Table S7A. Sensitivity analysis of neurological/severity-score redundancy.**

| **Model** | **No. predictors** | **AUC** | **AUPRC** | **Brier** | **Accuracy** | **Sensitivity** | **Specificity** | **PPV** | **NPV** | **F1** | **Threshold** | **AUC 95% CI** |
| --- | --- | --- | --- | --- | --- | --- | --- | --- | --- | --- | --- | --- |
| Model A: Primary full RF model | 65 | 0.963 | 0.914 | 0.122 | 0.903 | 0.875 | 0.914 | 0.800 | 0.949 | 0.836 | 0.450 | 0.929-0.989 |
| Model B: PCA-neuroseverity RF model | 61 | 0.951 | 0.911 | 0.125 | 0.929 | 0.844 | 0.963 | 0.900 | 0.940 | 0.871 | 0.450 | 0.902-0.987 |
| Model C: Reduced neurological-score RF model | 62 | 0.963 | 0.927 | 0.124 | 0.920 | 0.906 | 0.926 | 0.829 | 0.962 | 0.866 | 0.450 | 0.929-0.989 |

Model A used the primary predictor set; Model B replaced NRS-2002, APACHE II, GCS, mRS, and NIHSS with Neuroseverity_PC1; Model C retained a reduced neurological-score set.

**Supplementary Table S7B. PCA explained variance for the neurological/severity-score cluster.**

| **Component** | **Explained variance ratio** |
| --- | --- |
| PC1 | 0.408 |
| PC2 | 0.183 |
| PC3 | 0.158 |
| PC4 | 0.141 |
| PC5 | 0.110 |

**Supplementary Table S7C. PC1 loadings for the neurological/severity-score cluster.**

| **Feature** | **PC1 loading** | **Absolute loading** |
| --- | --- | --- |
| NRS2002 | 0.359 | 0.359 |
| APACHE2 | 0.412 | 0.412 |
| GCS | -0.473 | 0.473 |
| MRS | 0.464 | 0.464 |
| NIHSS | 0.512 | 0.512 |

**Supplementary Table S8. SHAP feature-rank stability across bootstrap resampling (top 20 features).**

| **Feature** | **Mean rank** | **Median rank** | **Rank IQR** | **Top-5 frequency** | **Top-10 frequency** | **Top-20 frequency** | **Times in top 20** |
| --- | --- | --- | --- | --- | --- | --- | --- |
| CRP | 1.28 | 1.00 | 0.00 | 1.00 | 1.00 | 1.00 | 100 |
| ALB | 2.72 | 2.00 | 1.00 | 0.96 | 1.00 | 1.00 | 100 |
| Enteral_Infusion_Rate | 3.30 | 3.00 | 2.00 | 0.93 | 1.00 | 1.00 | 100 |
| MRS | 5.03 | 5.00 | 2.25 | 0.65 | 0.99 | 1.00 | 100 |
| NRS2002 | 5.09 | 5.00 | 3.00 | 0.61 | 0.99 | 1.00 | 100 |
| APACHE2 | 5.72 | 6.00 | 2.00 | 0.43 | 1.00 | 1.00 | 100 |
| NIHSS | 6.26 | 6.00 | 2.00 | 0.29 | 1.00 | 1.00 | 100 |
| CV | 10.34 | 9.00 | 4.00 | 0.05 | 0.57 | 0.91 | 91 |
| NEUT | 12.71 | 12.00 | 5.50 | 0.00 | 0.25 | 0.84 | 84 |
| Oxygen_Therapy | 10.13 | 10.00 | 4.00 | 0.04 | 0.52 | 0.83 | 83 |
| LYM | 13.77 | 14.00 | 5.75 | 0.00 | 0.17 | 0.78 | 78 |
| Daily_Potassium_Dose | 11.33 | 11.00 | 4.00 | 0.03 | 0.36 | 0.76 | 76 |
| tHb | 13.53 | 13.50 | 5.75 | 0.01 | 0.19 | 0.74 | 74 |
| Analgesic_Agent | 13.07 | 12.00 | 6.00 | 0.00 | 0.20 | 0.69 | 69 |
| WBC | 13.72 | 13.00 | 6.00 | 0.00 | 0.14 | 0.67 | 67 |
| HGB | 14.41 | 15.00 | 5.00 | 0.00 | 0.10 | 0.64 | 64 |
| Glu | 13.97 | 14.00 | 6.00 | 0.00 | 0.11 | 0.60 | 60 |
| GCS | 15.23 | 15.00 | 4.00 | 0.00 | 0.04 | 0.60 | 60 |
| MAP | 14.47 | 14.50 | 5.00 | 0.00 | 0.05 | 0.58 | 58 |
| Lac | 13.91 | 14.00 | 6.00 | 0.00 | 0.12 | 0.54 | 54 |

Frequencies indicate the proportion of bootstrap iterations in which a feature appeared among the top-ranked SHAP contributors.

**Supplementary Table S9A. Full model, study-specific 12-predictor model, Liao-compatible overlapping-predictor model, and parsimonious logistic-regression comparison.**

| **Model** | **Model type** | **No. predictors** | **AUC** | **AUPRC** | **Brier** | **Accuracy** | **Sensitivity** | **Specificity** | **PPV** | **NPV** | **F1** | **Threshold** | **AUC 95% CI** |
| --- | --- | --- | --- | --- | --- | --- | --- | --- | --- | --- | --- | --- | --- |
| Primary full RF model | Random forest | 65 | 0.963 | 0.914 | 0.122 | 0.903 | 0.875 | 0.914 | 0.800 | 0.949 | 0.836 | 0.450 | 0.929-0.988 |
| Study-specific 12-predictor RF model | Random forest | 12 | 0.963 | 0.923 | 0.090 | 0.903 | 0.875 | 0.914 | 0.800 | 0.949 | 0.836 | 0.450 | 0.925-0.992 |
| Liao-compatible overlapping-predictor model | Random forest | 9 | 0.900 | 0.858 | 0.135 | 0.841 | 0.812 | 0.852 | 0.684 | 0.920 | 0.743 | 0.450 | 0.825-0.959 |
| Study-specific 12-predictor regularized logistic regression | Regularized logistic regression | 12 | 0.973 | 0.948 | 0.057 | 0.903 | 0.938 | 0.889 | 0.769 | 0.973 | 0.845 | 0.205 | 0.942-0.994 |

The Liao-compatible model used only predictors that could be mapped to the present dataset. Exact replication of the Liao et al. 12-predictor model was not possible because several predictors were not collected.

**Supplementary Table S9B. Mapping of the Liao et al. 12 predictors to available variables in the present cohort.**

| **Liao predictor** | **Mapped feature(s) in this cohort** | **Availability status** | **Used in Liao-compatible model** |
| --- | --- | --- | --- |
| Probiotics |  | not collected / not found | No |
| Antibiotics | Antibiotic_Therapy | available in final analytic CSV and used if selected | Yes |
| ENType | Enteral_Formula_Type | available in final analytic CSV and used if selected | Yes |
| Room temperature |  | not collected / not found | No |
| Sodium | Na | available in final analytic CSV and used if selected | Yes |
| Potassium | K | available in final analytic CSV and used if selected | Yes |
| CRP | CRP | available in final analytic CSV and used if selected | Yes |
| Age | Age | available in final analytic CSV and used if selected | Yes |
| PCT |  | not collected / not found | No |
| Rate | Enteral_Infusion_Rate | available in final analytic CSV and used if selected | Yes |
| Warmer |  | not collected / not found | No |
| ENStart | Fasting_Within_24h; Fasting_24h_to_48h | available in final analytic CSV and used if selected | Yes |

ENStart was approximated by available fasting-duration indicators. Room temperature, probiotics, PCT, and warmer use were not collected in the final analytic dataset.

**4. Supplementary figures**

**Supplementary Figure S1A. Missingness bar plot for variables with missing values in the final analytic dataset.**


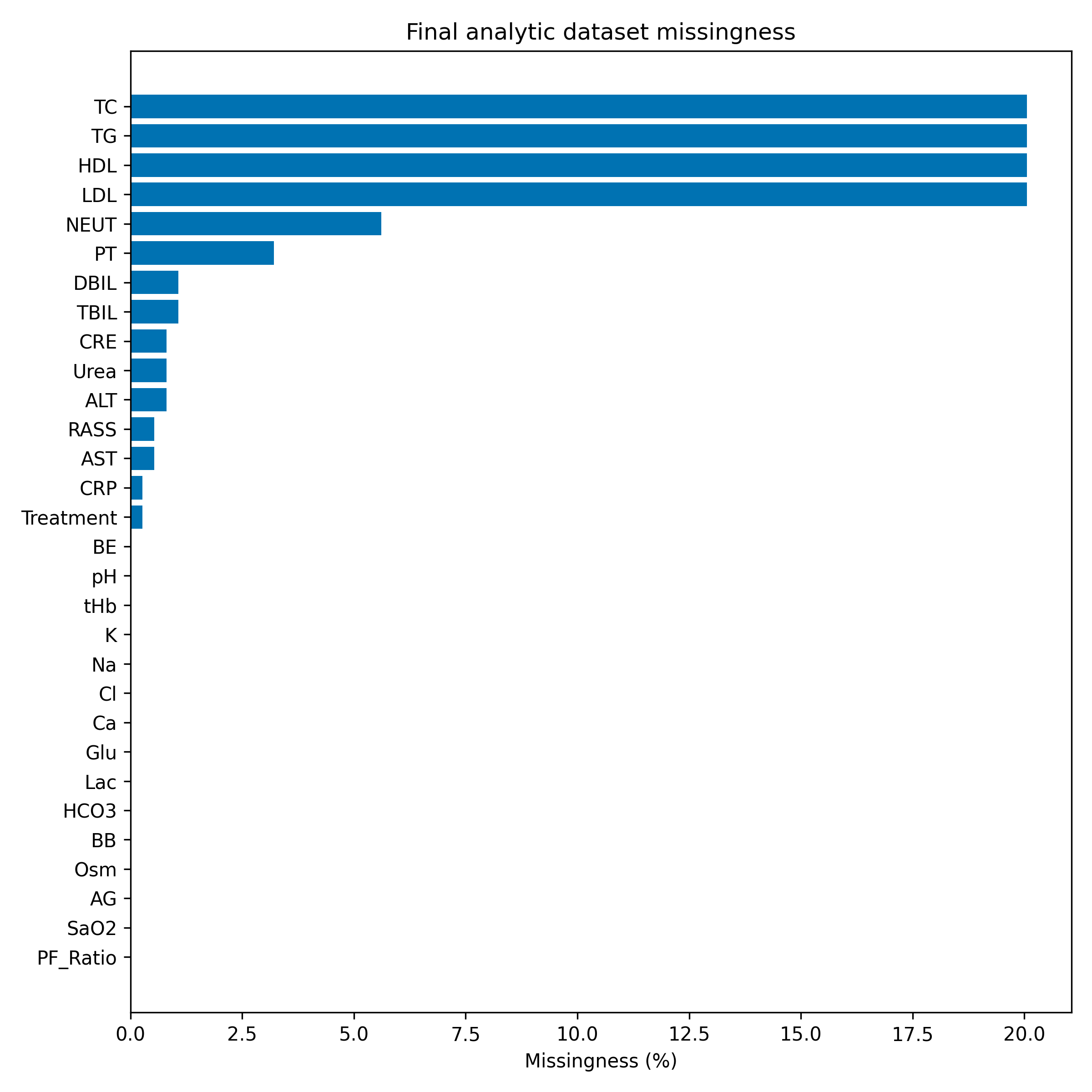


**Supplementary Figure S1B. Missingness heatmap for variables with missing values in the final analytic dataset.**


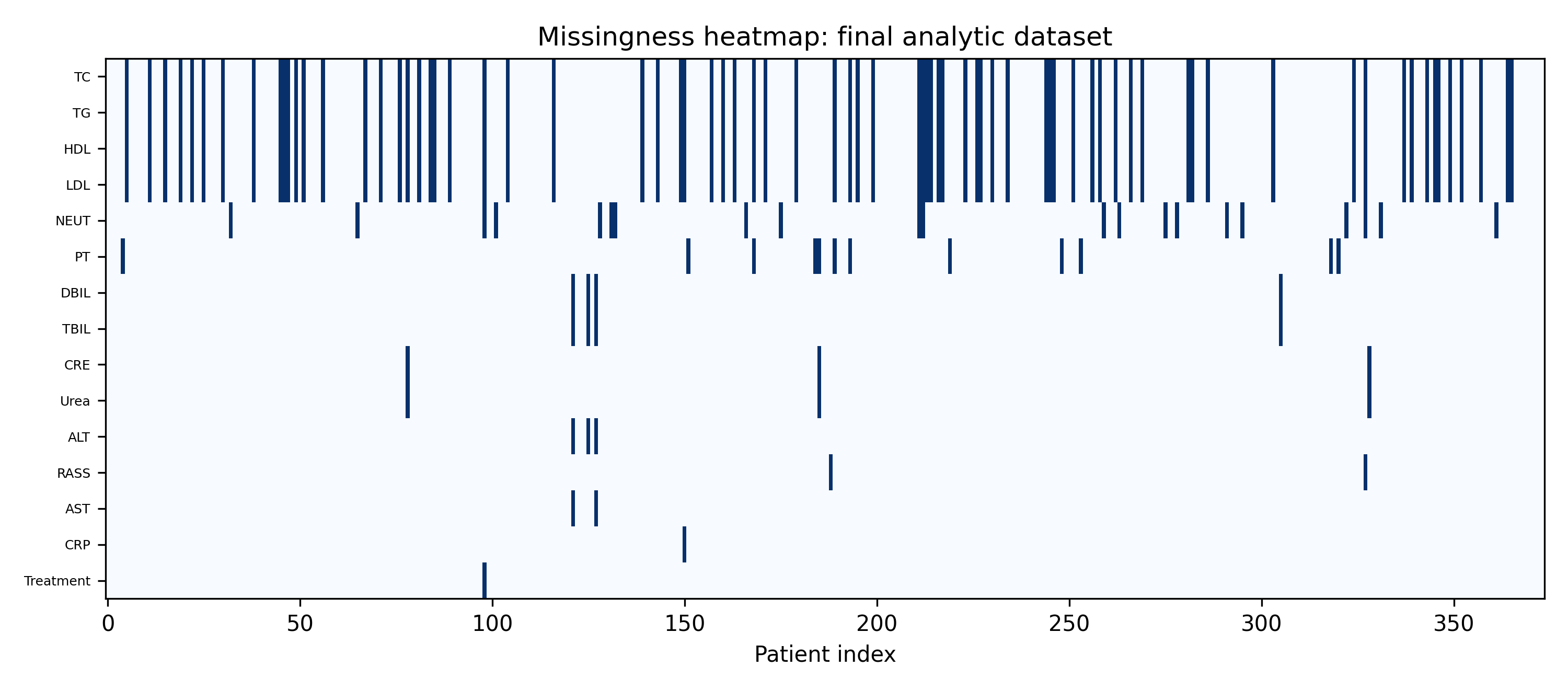


**Supplementary Figure S2A. Scatter plot comparing HGB with tHb×10.**


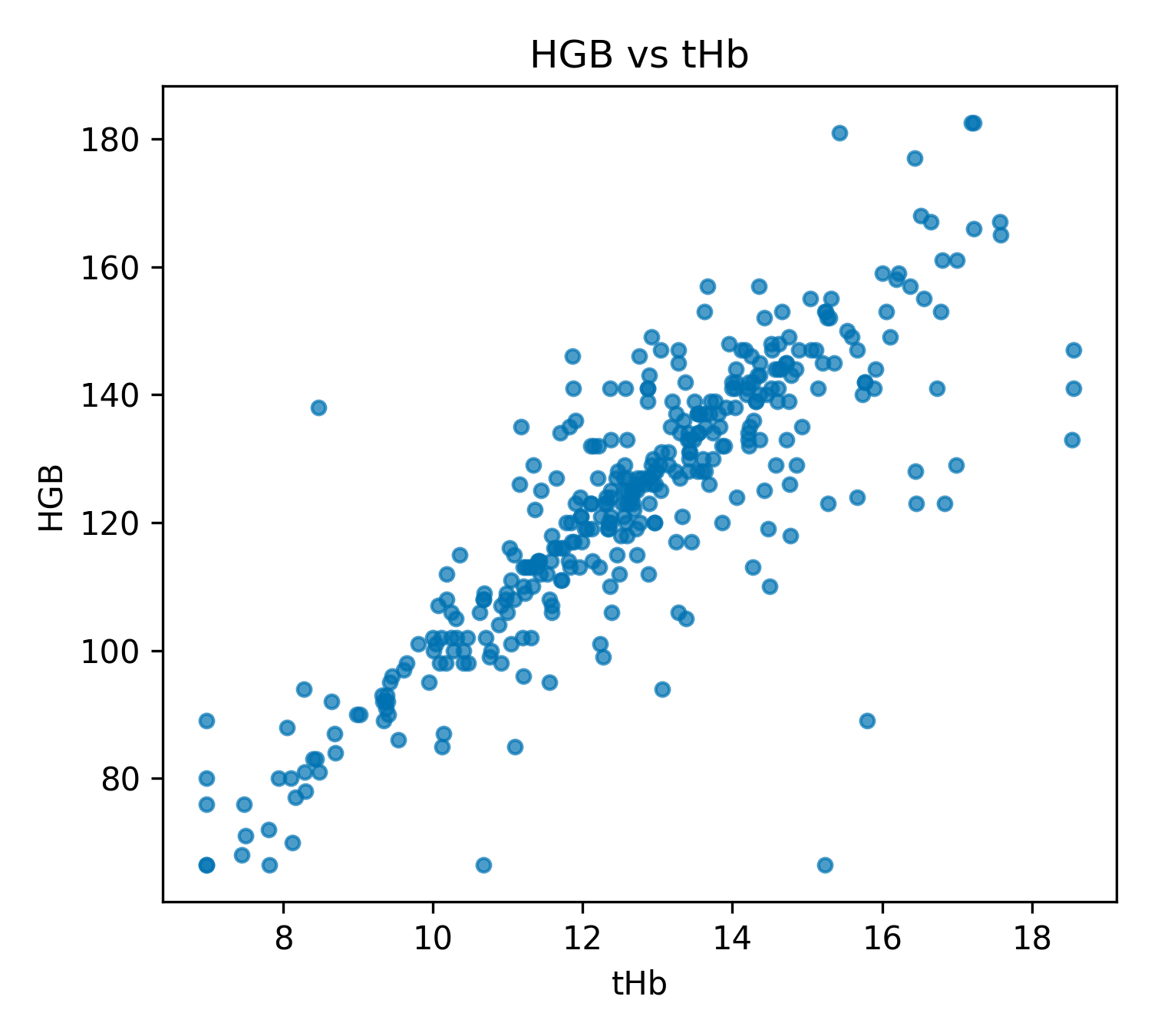


**Supplementary Figure S2B. Difference plot for HGB - tHb×10.**


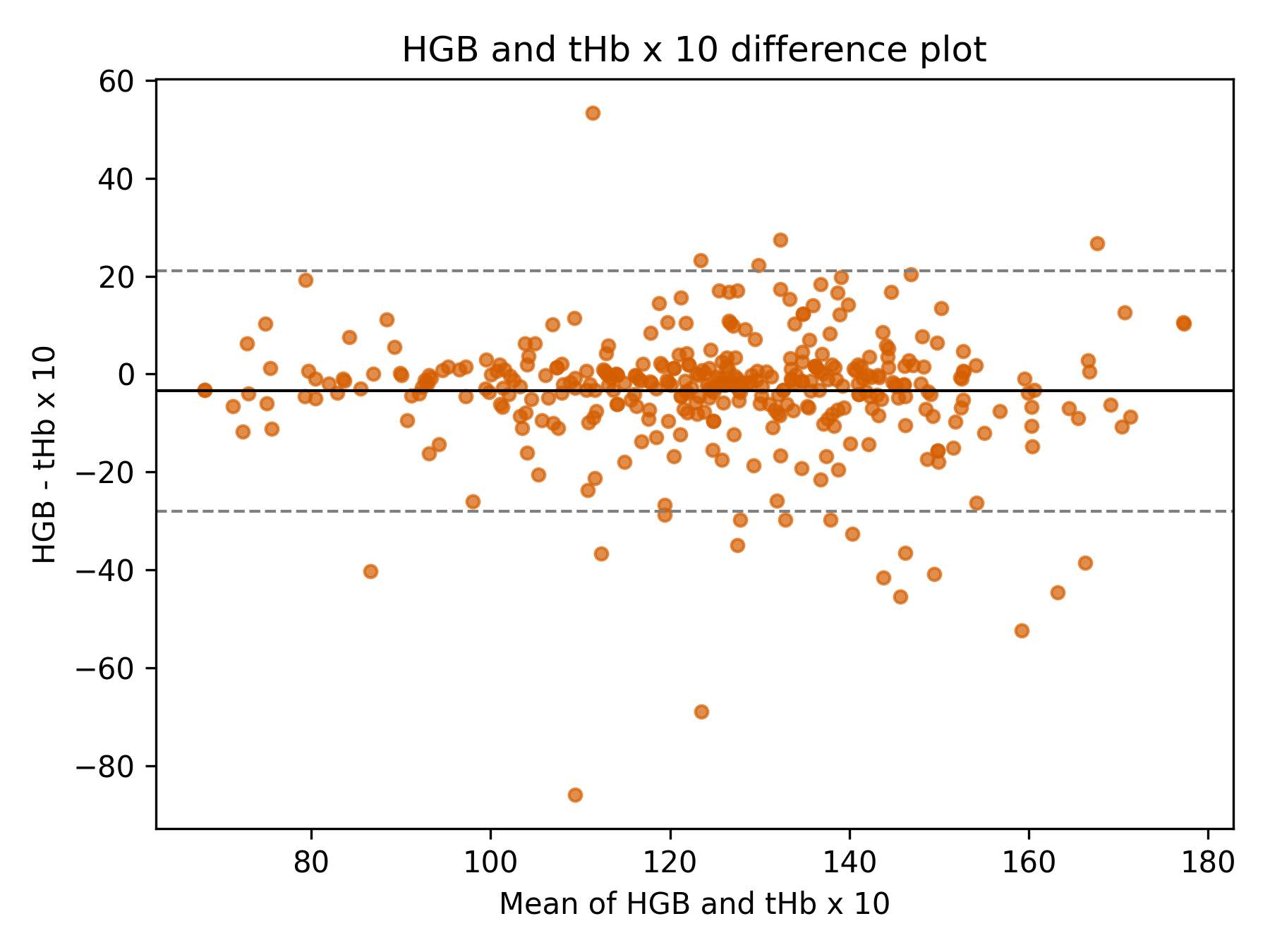


**Supplementary Figure S3. Spearman correlation heatmap among selected predictors.**


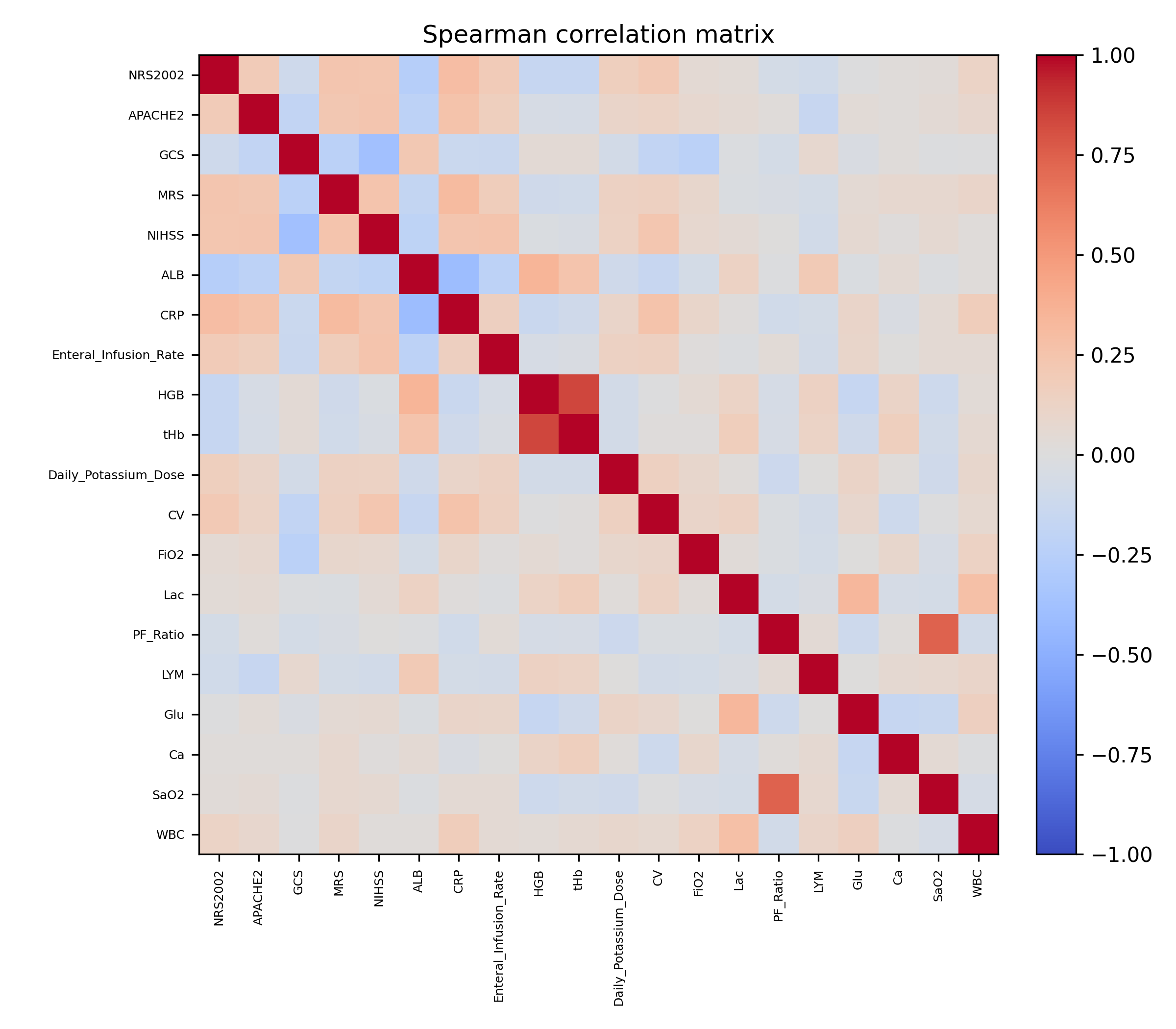


**Supplementary Figure S4. VIF bar plot for selected predictors.**


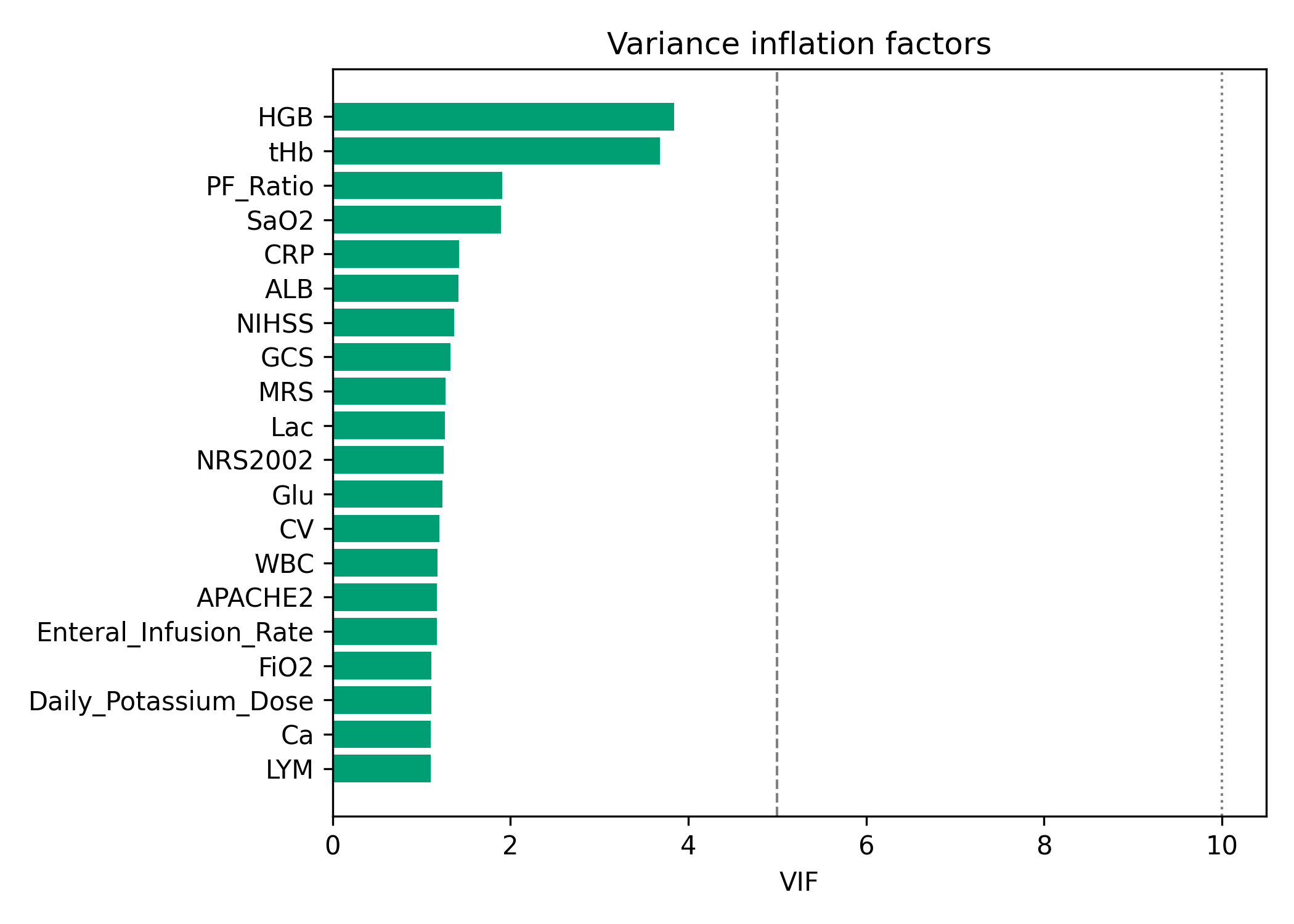


**Supplementary Figure S5A. PCA scree plot for the neurological/severity-score cluster.**


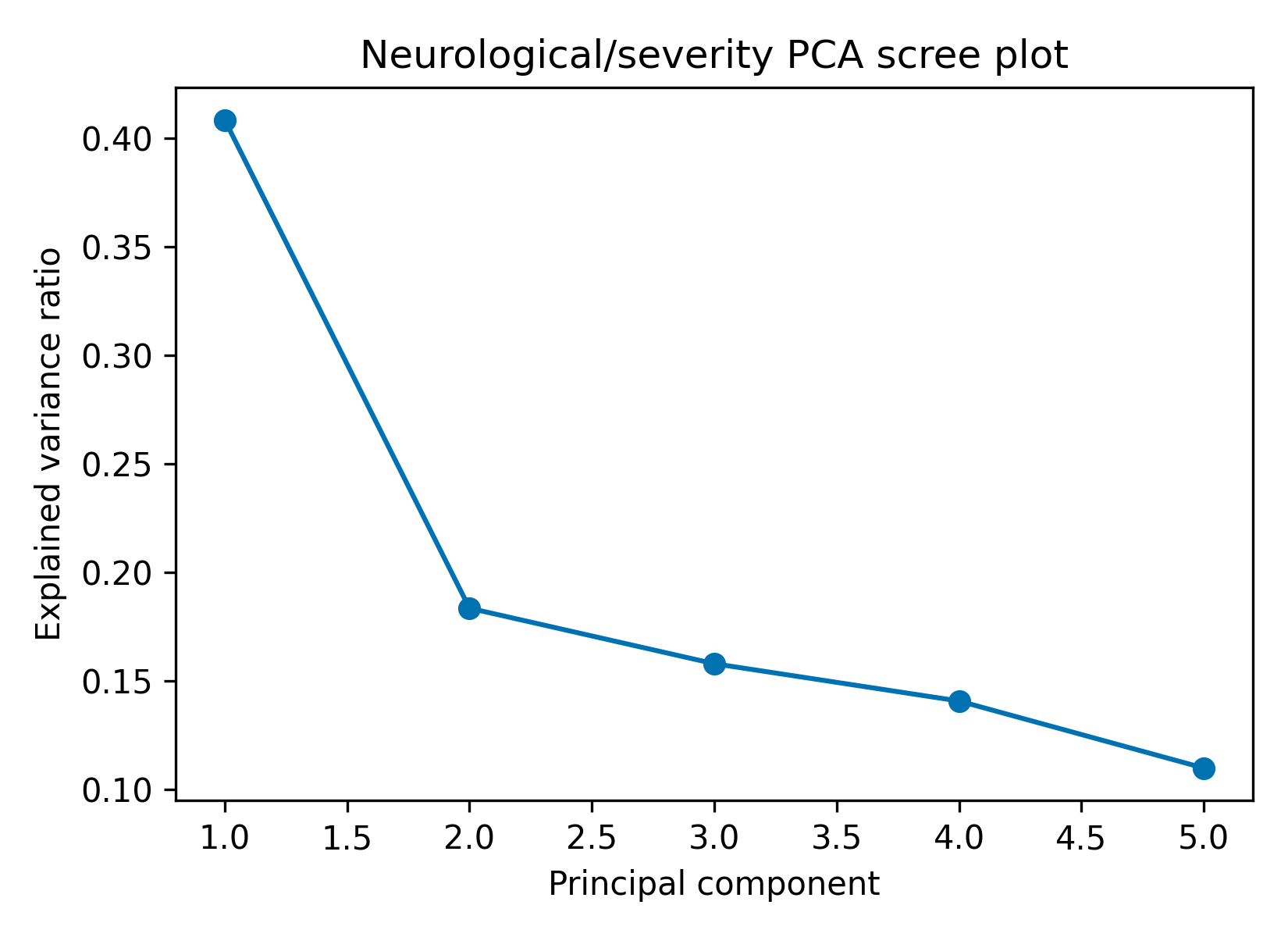


**Supplementary Figure S5B. PC1 loadings for the neurological/severity-score cluster.**


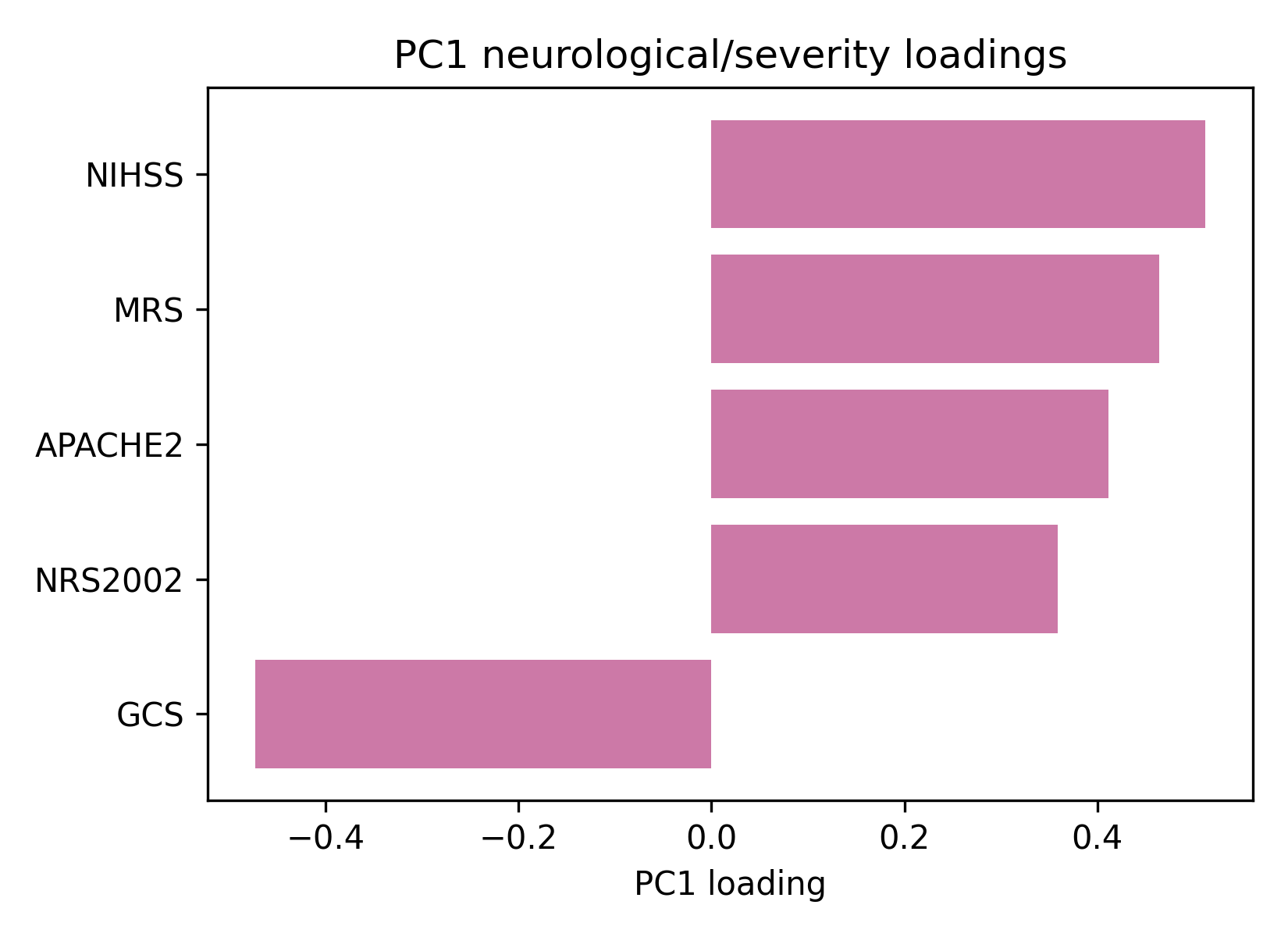


**Supplementary Figure S6A. ROC curves comparing the full, PCA-neuroseverity, and reduced neurological-score random forest models.**


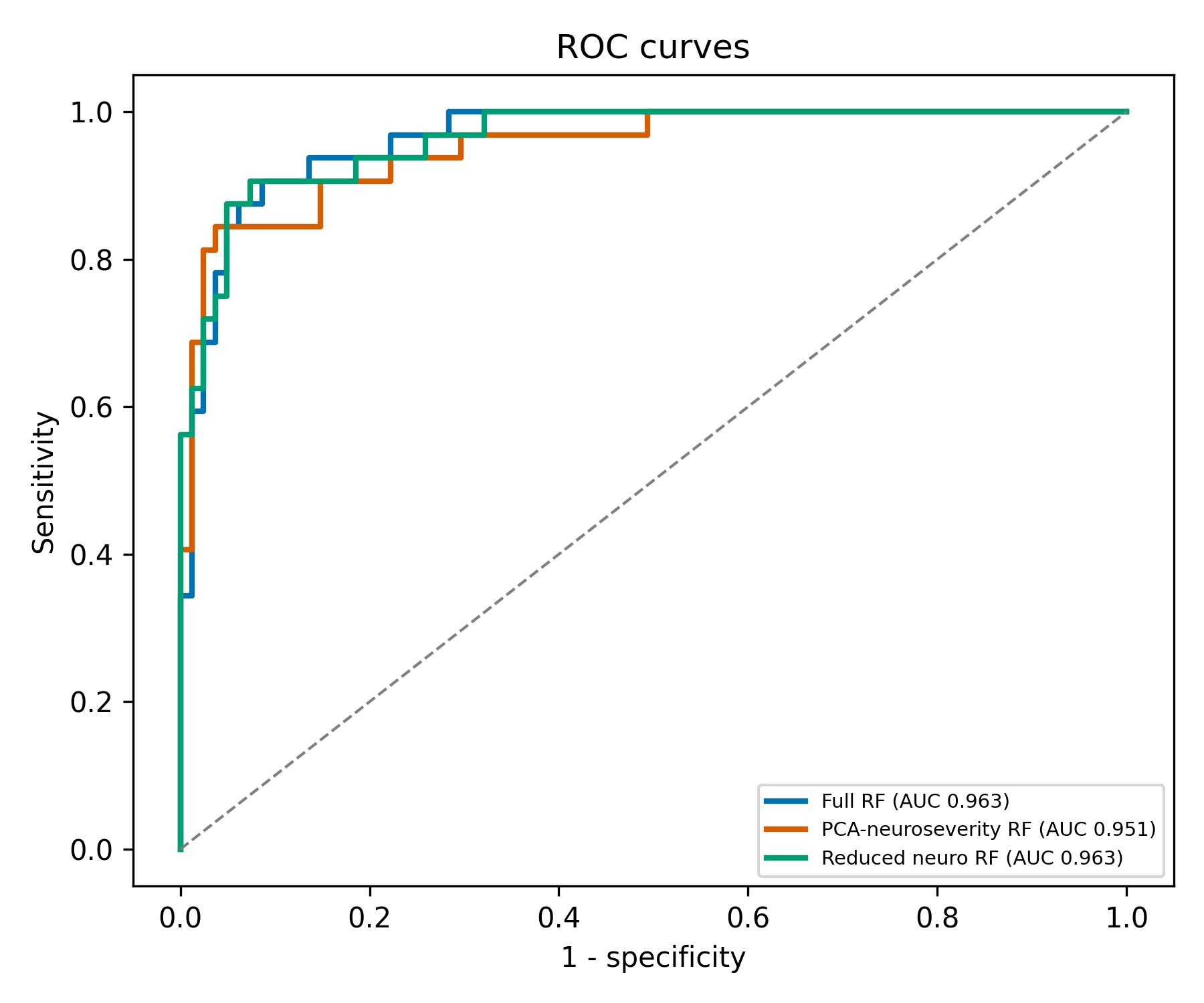


**Supplementary Figure S6B. Calibration curves comparing the full, PCA-neuroseverity, and reduced neurological-score random forest models.**


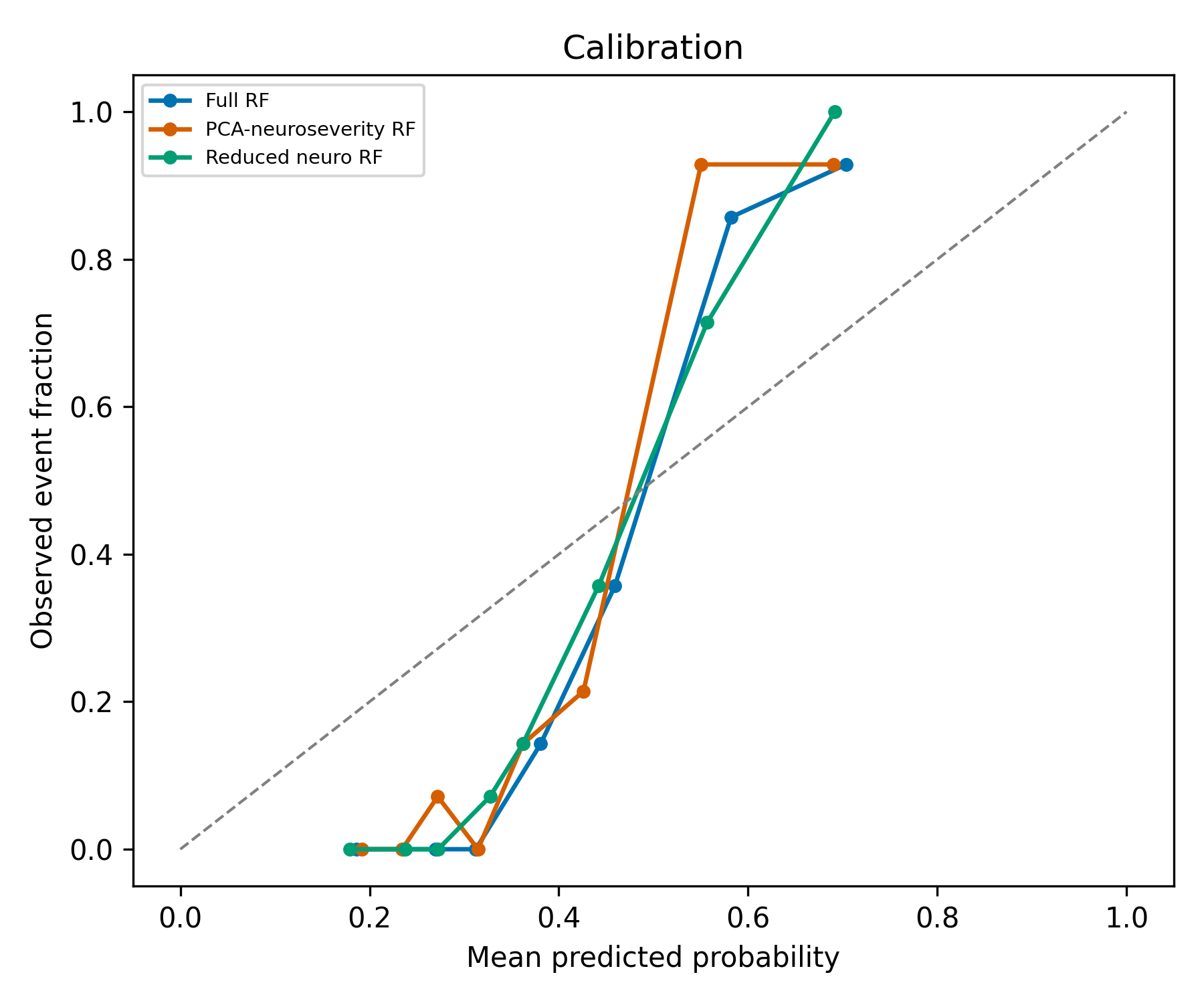


**Supplementary Figure S6C. Decision-curve analysis comparing the full, PCA-neuroseverity, and reduced neurological-score random forest models.**


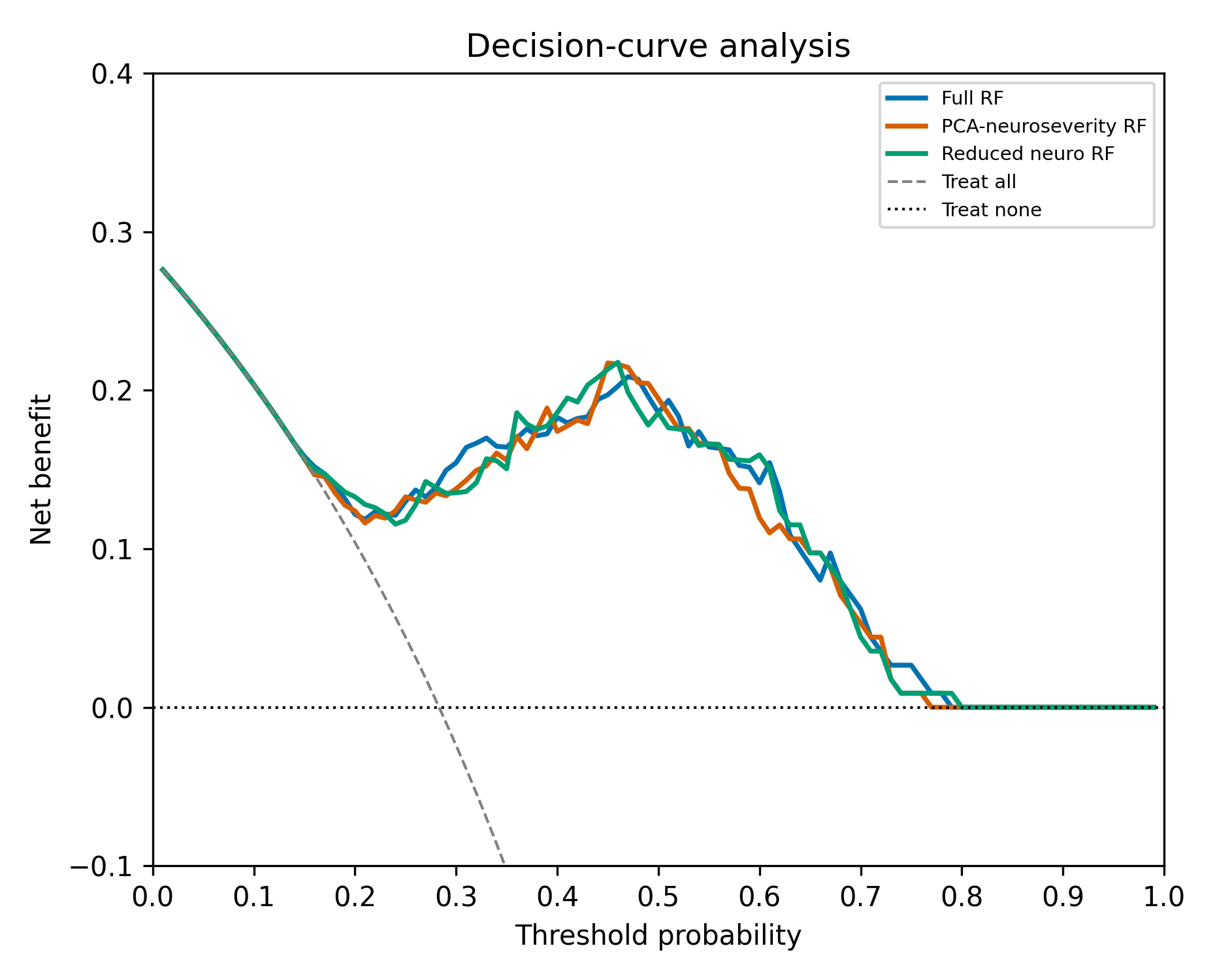


**Supplementary Figure S7A. Frequency of top-10 SHAP rank membership across bootstrap resampling.**


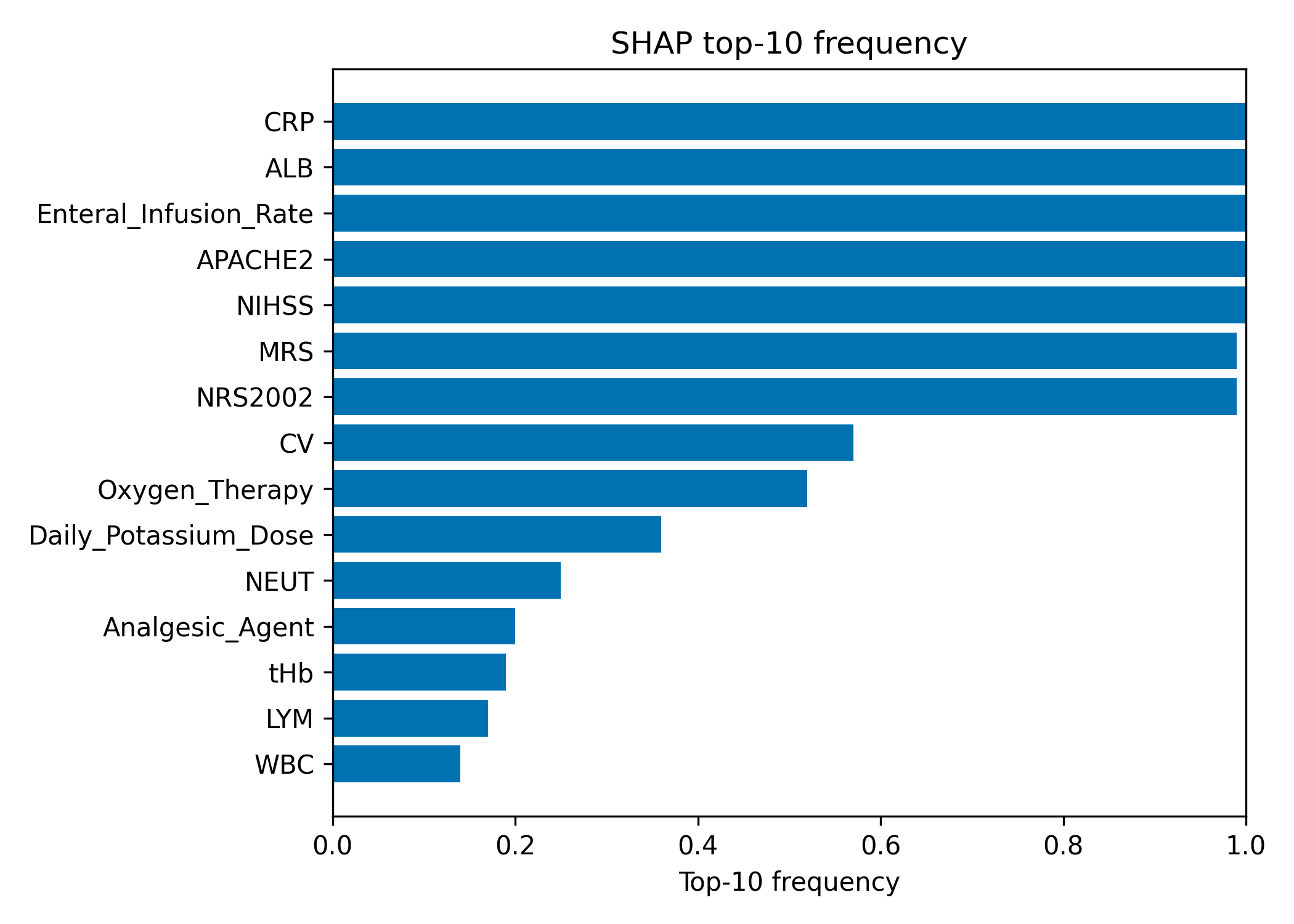


**Supplementary Figure S7B. SHAP rank-stability heatmap across bootstrap resampling.**


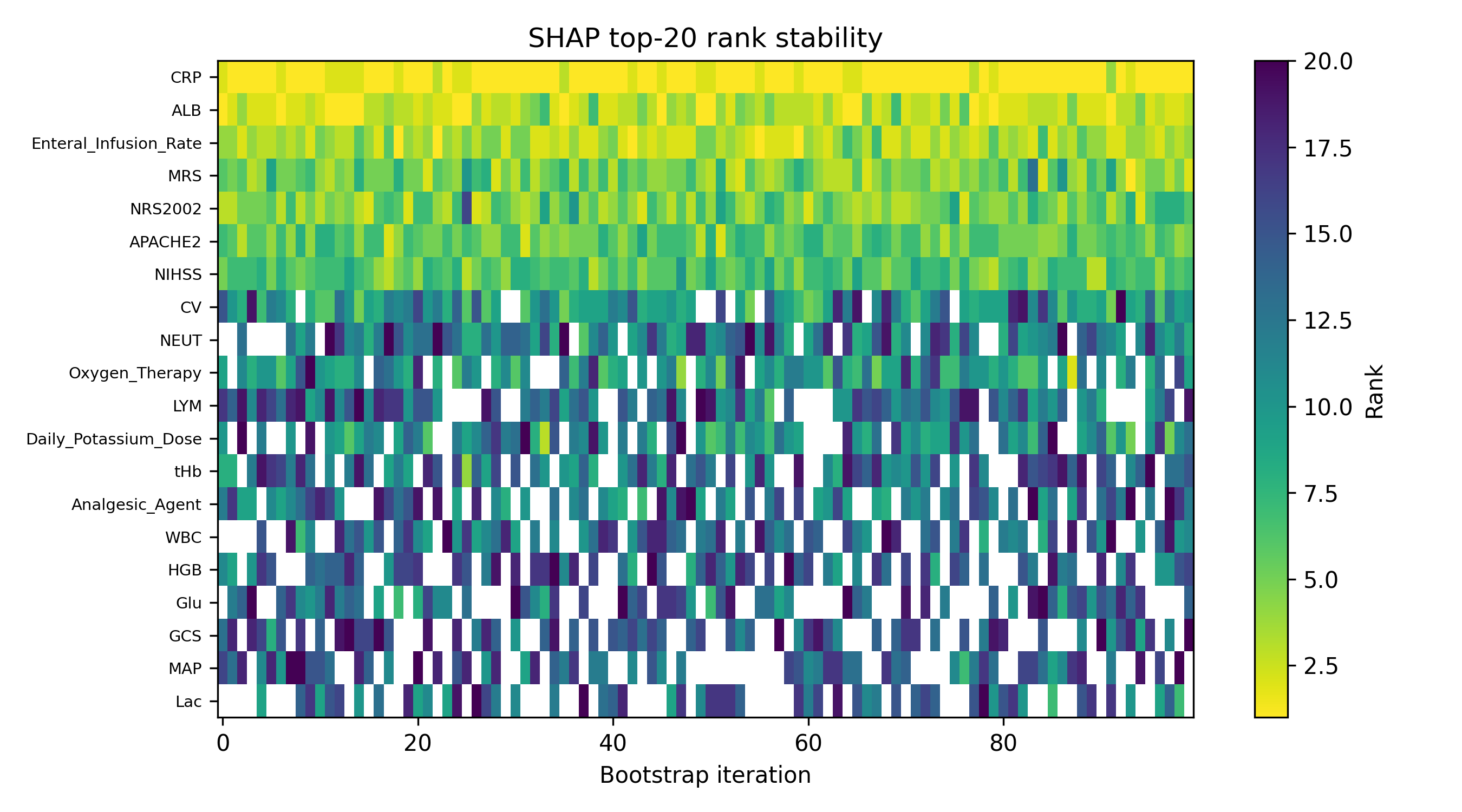


**Supplementary Figure S8A. Feature-overlap mapping for the Liao-compatible model.**


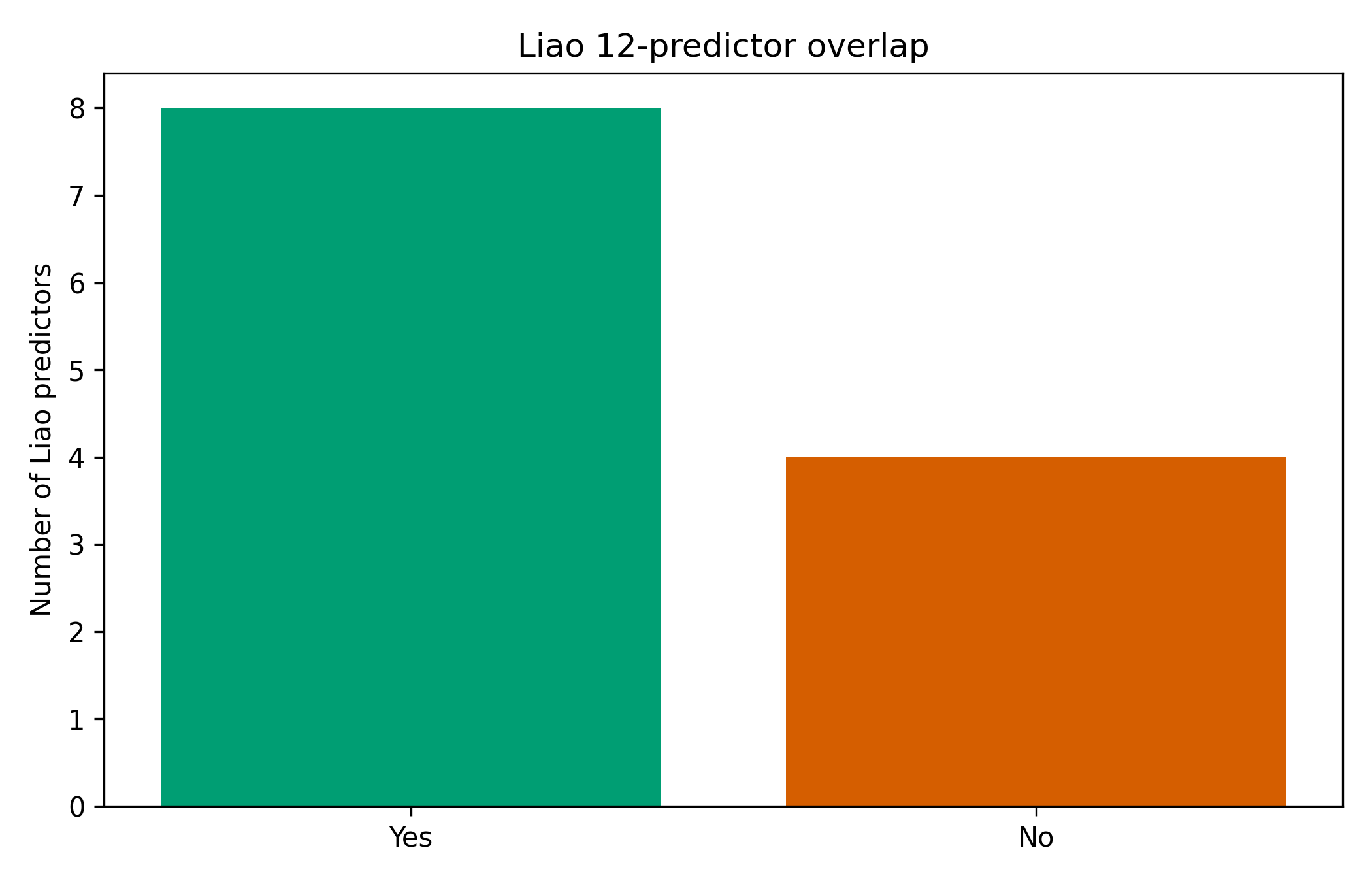


**Supplementary Figure S8B. ROC curves comparing the full, study-specific 12-predictor, Liao-compatible, and parsimonious logistic-regression models.**


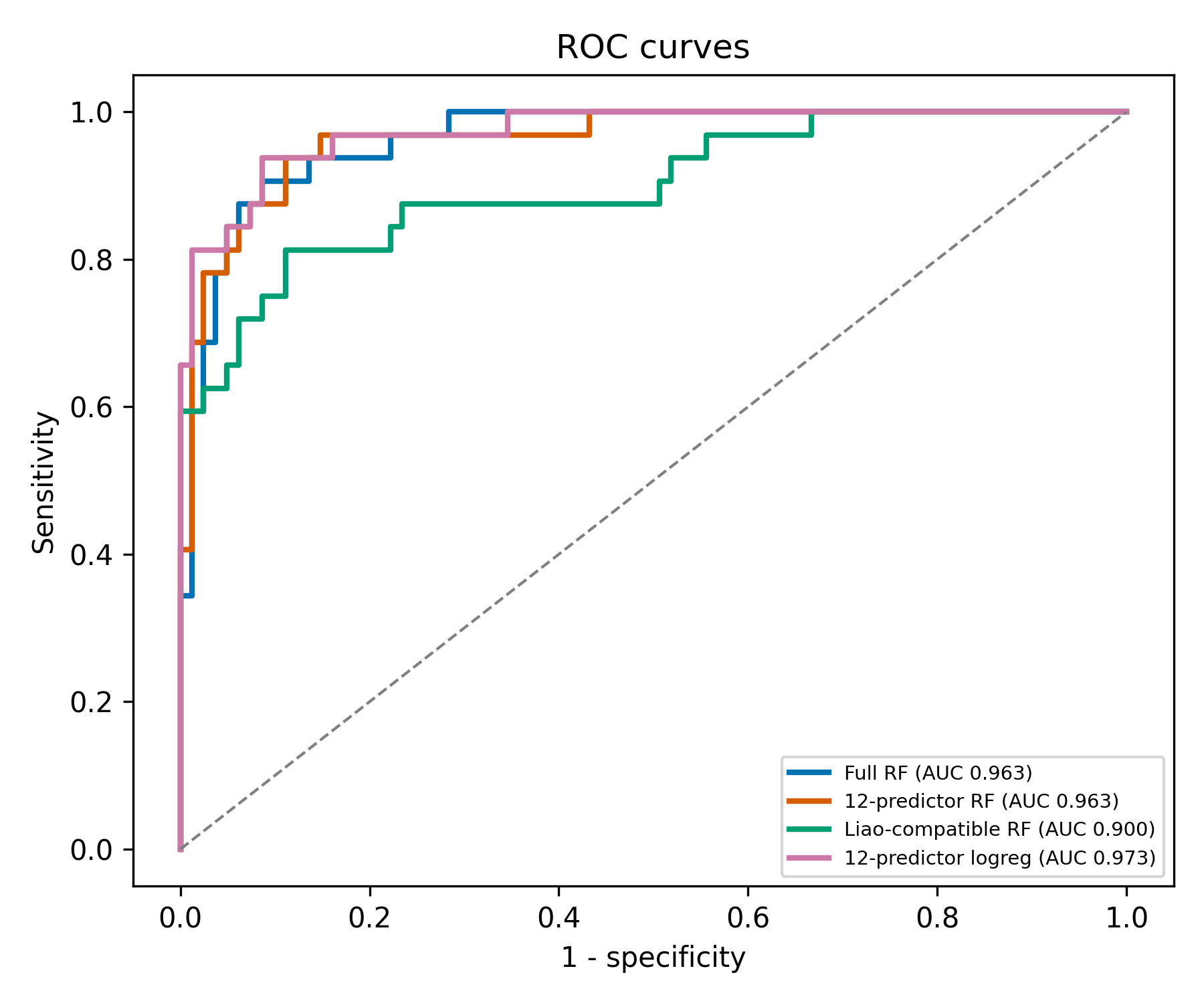


**Supplementary Figure S8C. Calibration curves comparing the full, study-specific 12-predictor, Liao-compatible, and parsimonious logistic-regression models.**


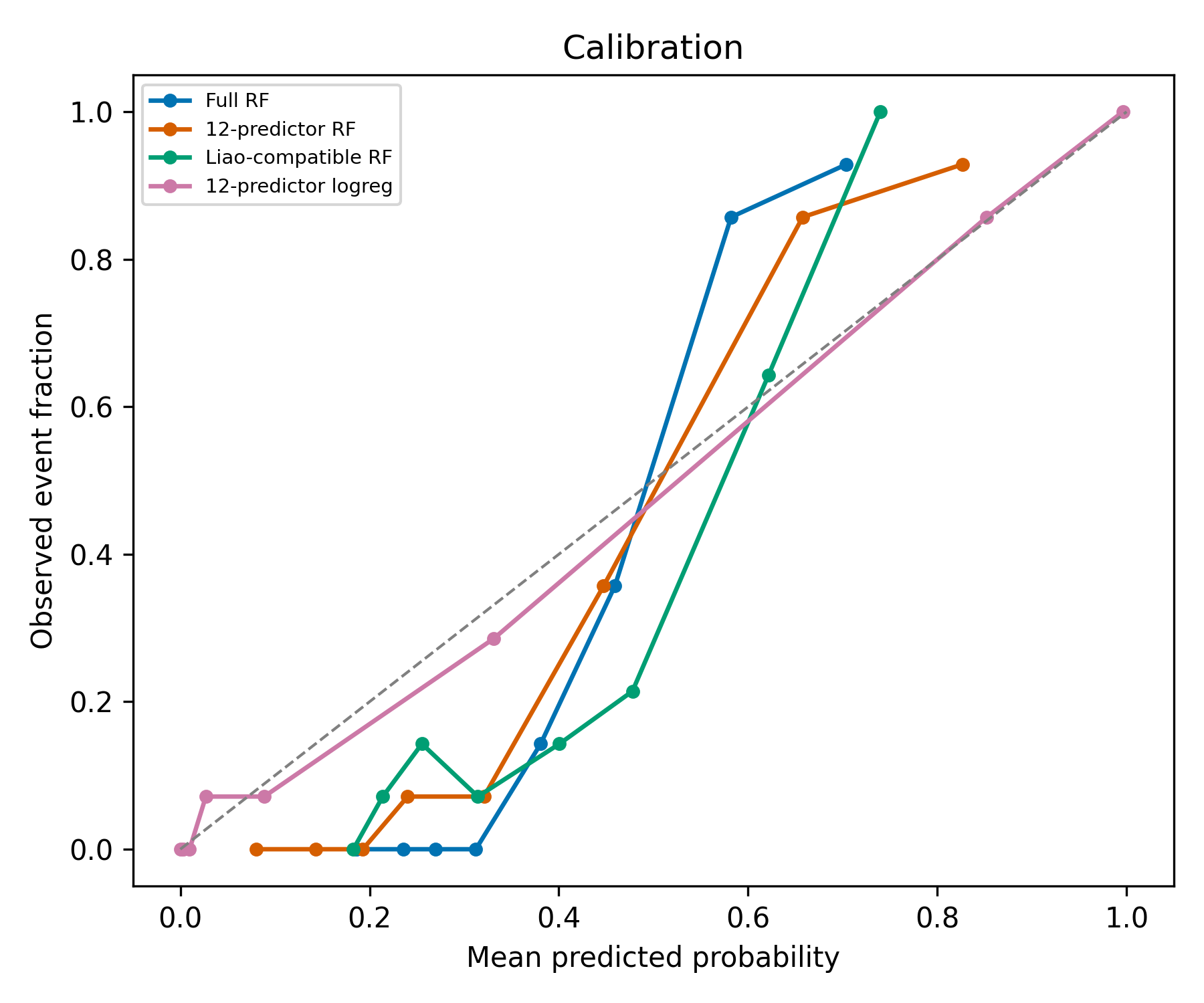


**Supplementary Figure S8D. Decision-curve analysis comparing the full, study-specific 12-predictor, Liao-compatible, and parsimonious logistic-regression models.**


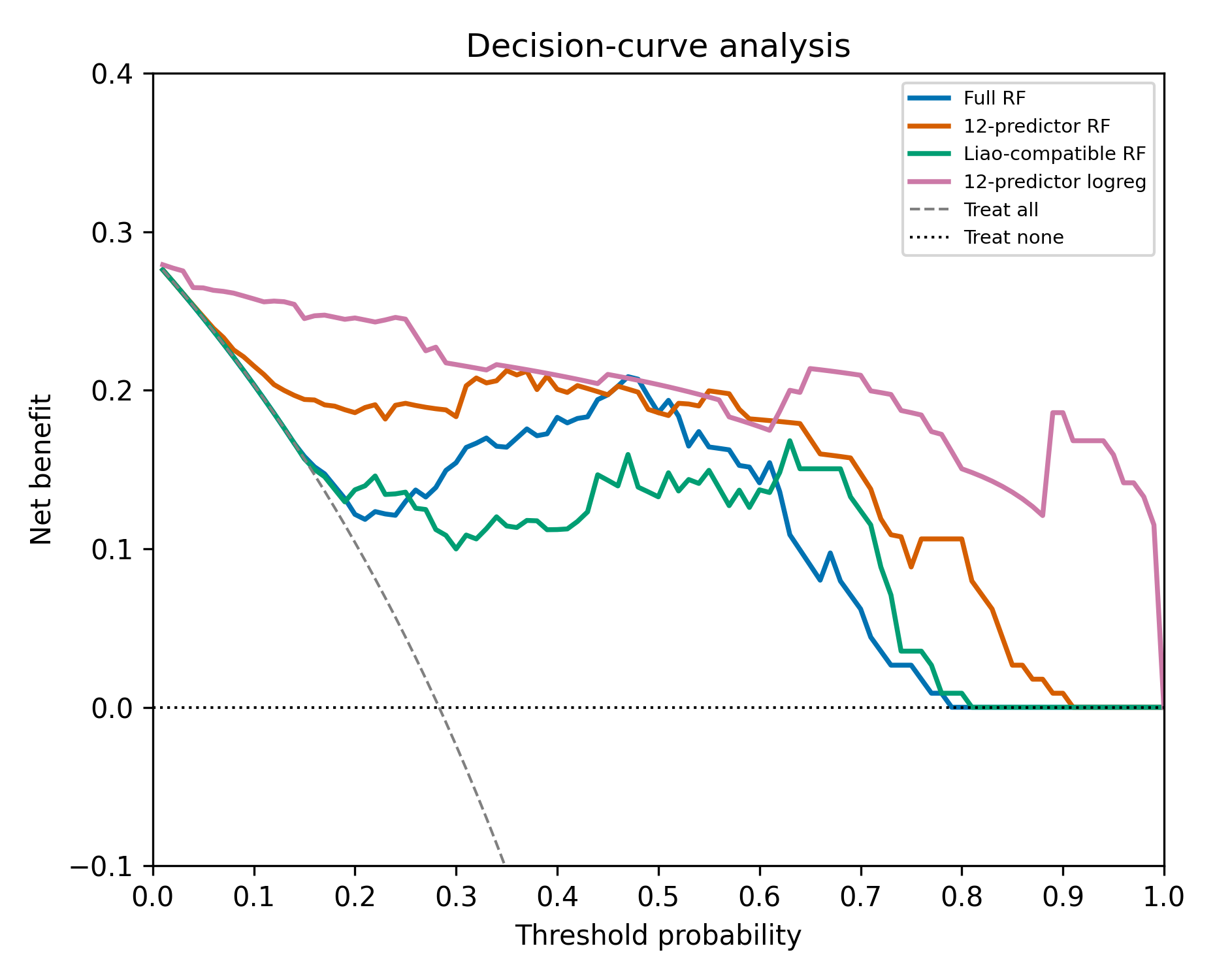

Supplement: Supplementary file 1 [file Table_1.docx]
